# Correction: Dual pathway for metabolic engineering of *Escherichia coli* to produce the highly valuable hydroxytyrosol

**DOI:** 10.1371/journal.pone.0226760

**Published:** 2019-12-13

**Authors:** The PLOS ONE Staff

## Notice of republication

This article was republished on December 5, 2019 to correct errors in the Funding statement and to add an ORCID iD for the last author. The publisher apologizes for these errors. Please download this article again to view the correct version. The originally published, uncorrected article and the republished, corrected article are provided here for reference.

## Supporting information

S1 File Originally published, uncorrected article. (PDF)

S2 File Republished corrected article. (PDF)
