# Supplementary material for: Dual pathway for metabolic engineering of Escherichia coli to produce the highly valuable hydroxytyrosol
Source: PLoS One. 2019 Nov 4;14(11):e0212243. doi: 10.1371/journal.pone.0212243 (PMC6828502; doi:10.1371/journal.pone.0212243)
Supplement: S1 Table — (DOCX) [file pone.0212243.s001.docx]

**Table S1: List of primers used for gene clonings included in this study.**

| Gene | Oligo sequence name | Direction | Oligo sequence (5’🡪3’) | NCBI code |
| --- | --- | --- | --- | --- |
| *ALR-K, Aldehyde reductase* | yahK-fw1 | upstream | CCATGGGCAAGATCAAAGCTGTTGGTGCATATTC | KT334551 |
|  | yahK-rv1 | downstream | ACAGTCTGCAGGAGAGCGAAGTTAATATGTTAAACCACAG |  |
| *ALR-D, Aldehyde reductase* | yqhD-fw1 | upstream | ACAGATCCATGGGCAACAACTTTAATCTGCACACC | KT334550 |
|  | yqhD-rv1 | downstream | AGACATCTGCAGGGACGAAATGCCCGAAAAC |  |
| *AAS, Aromatic Acetaldehyde Synthase* | PcTDC2bfw | upstream | GTTACCATGGGCTCCATCGATAATCTTACTG | KT334544 |
|  | PcTDC2bfw2 | downstream | AAAGGATCCCTCAAACCAGAGCAGAGACATG |  |
| *TYR, Tyrosinase* | RsTYRfw2 | upstream | ATAGGACATATGGTCGTGCGTAGAAC | KT334543 |
|  | RsTYRrv2 | downstream | AATCTCGAGCAATCCATCAATCAAAT |  |
