# Supplementary figures and images for: Dual pathway for metabolic engineering of Escherichia coli to produce the highly valuable hydroxytyrosol
Source: PLoS One. 2019 Nov 4;14(11):e0212243. doi: 10.1371/journal.pone.0212243 (PMC6828502; doi:10.1371/journal.pone.0212243)

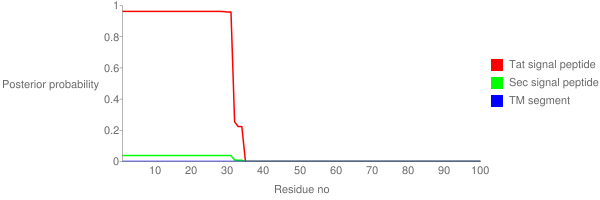

Supplement: S1 Fig — The PRED-TAT online software was utilized. The cleavage site was predicted between the two alanines (in bold) of AVAAD. (TIF) [file pone.0212243.s002.tif]

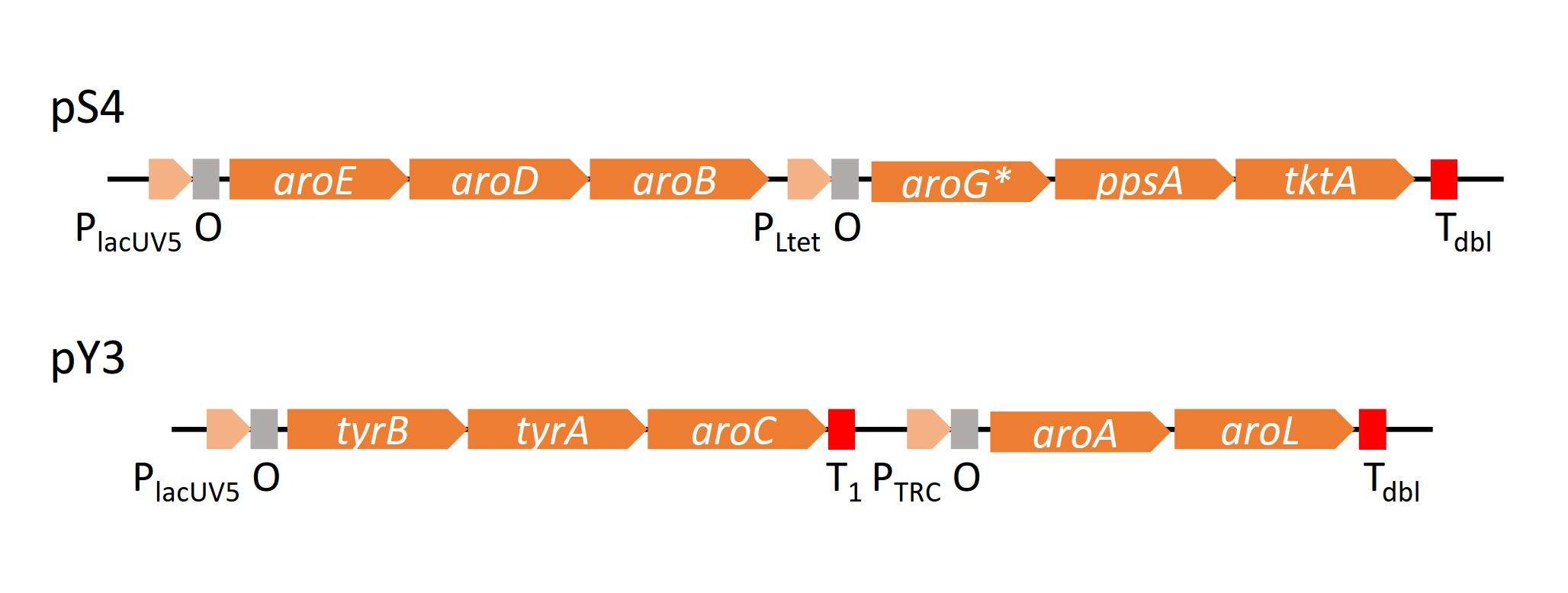

Supplement: S2 Fig — AroA, 5-enolpyruvoylshikimate 3-phosphate synthase; AroB, Dehydroquinate synthase; AroC, chorismate synthase; AroD, Dehydroquinate dehydratase; AroE, shikimate dehydrogenase; AroG, 3-deoxy-D-arabino-heptulosonate synthase; AroL, shikimate kinase; PpsA, phosphoenolpyruvate synthase; TktA, transketolase A; TyrA, chorismate mutase/prephenate dehydrogenase; TyrB, tyrosine aminotransferase; P, Promoter; O, Operator. T, Termination sequence [29]. (TIF) [file pone.0212243.s003.tif]

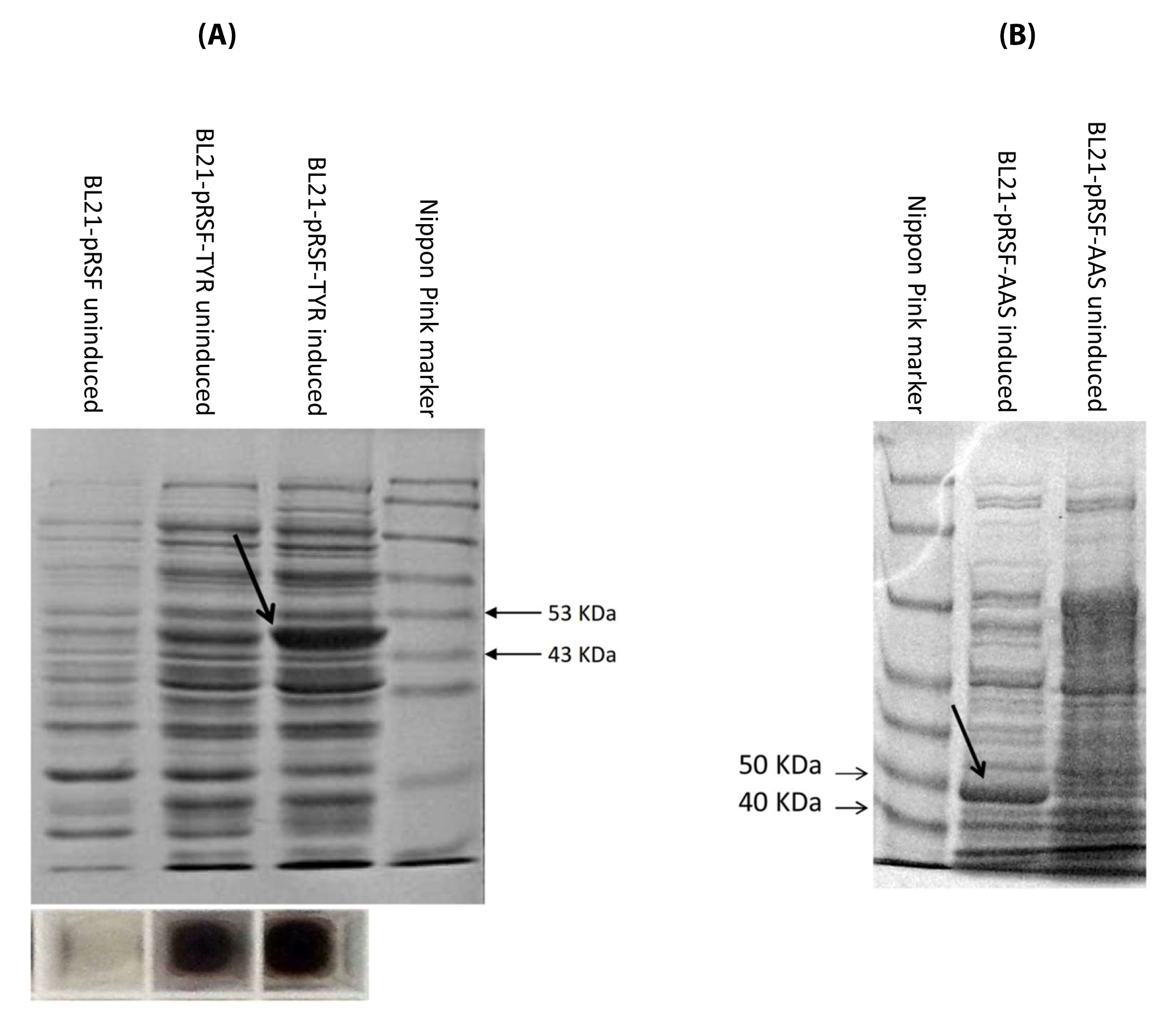

Supplement: S3 Fig — (A) Upper part, protein expression in Escherichia coli to certify the tyrosinase protein expression. In the first lane total proteins from E. coli BL21-pRSF in non-induced conditions were loaded. In the second, total proteins from BL21-pRSF-RsTYR in non-induced conditions were loaded while in the third lane total proteins from BL21-pRSF-RsTYR in induced conditions were loaded. In the last lane the PiNK prestained protein ladder was loaded. The arrow in the protein marker helps to estimate the size of the protein band. Lower part, colorimetric assay with the protein crude extracts to assess the activity of tyrosinase as described in Material and Methods. (B) Protein expression in E. coli to certify the AAS expression. In the first lane the PiNK prestained protein ladder was loaded. In the second and the third lanes total proteins from BL21-pRSF-PcAAS in induced and non-induced conditions were loaded respectively. The arrows in the protein marker help to estimate the size of the expressed protein band. (TIF) [file pone.0212243.s004.tif]
