# Supplementary material for: Correction: Dual pathway for metabolic engineering of Escherichia coli to produce the highly valuable hydroxytyrosol
Source: PLoS One. 2019 Dec 13;14(12):e0226760. doi: 10.1371/journal.pone.0226760 (PMC6910702; doi:10.1371/journal.pone.0226760)
Supplement: S2 File — (PDF) [file pone.0226760.s002.pdf]

PLOS ONE

RESEARCH ARTICLE

Dual pathway for metabolic engineering of *Escherichia coli* to produce the highly valuable hydroxytyrosol

Emmanouil Trantas[1]*, **Eleni Navakoudis**[1], **Theofilos Pavlidis**[1], **Theodora Nikou**[2], **Maria Halabalaki**[2], **Leandros Skaltsounis**[2], **Filippos Ververidis**[1]*

1 Plant Biochemistry and Biotechnology Group, Laboratory of Biological and Biotechnological Applications, Department of Agriculture, School of Agricultural Sciences, Hellenic Mediterranean University, Heraklion, Greece, **2** Division of Pharmacognosy and Natural Product Chemistry, Department of Pharmacy, National and Kapodistrian University of Athens, Panepistimiopolis Zografou, Athens, Greece

* ververidis@hmu.gr (FV); mtrantas@hmu.gr (ET)

Check for updates

OPEN ACCESS

Citation: Trantas E, Navakoudis E, Pavlidis T, Nikou T, Halabalaki M, Skaltsounis L, et al. (2019) Dual pathway for metabolic engineering of *Escherichia coli* to produce the highly valuable hydroxytyrosol. PLoS ONE 14(11): e0212243. https://doi.org/10.1371/journal.pone.0212243

Editor: Marie-Joelle Virolle, Universite Paris-Sud, FRANCE

Received: January 25, 2019

Accepted: October 2, 2019

Published: November 4, 2019

Copyright: © 2019 Trantas et al. This is an open access article distributed under the terms of the Creative Commons Attribution License, which permits unrestricted use, distribution, and reproduction in any medium, provided the original author and source are credited.

Data Availability Statement: All relevant data are within the manuscript and its Supporting Information files.

Funding: This research has been co-financed by the European Regional Development Fund of the European Union and Greek national funds through the Operational Program Competitiveness, Entrepreneurship and Innovation, under the call "Research – Create – Innovate" (Project code: T1EDK-04267, by the acronym "ANTIOX-PLUS") awarded to FV. The funders had no role in study

Abstract

One of the most abundant phenolic compounds traced in olive tissues is hydroxytyrosol (HT), a molecule that has been attributed with a pile of beneficial effects, well documented by many epidemiological studies and thus adding value to products containing it. Strong antioxidant capacity and protection from cancer are only some of its exceptional features making it ideal as a potential supplement or preservative to be employed in the nutraceutical, agrochemical, cosmeceutical, and food industry. The HT biosynthetic pathway in plants (e.g. olive fruit tissues) is not well apprehended yet. In this contribution we employed a metabolic engineering strategy by constructing a dual pathway introduced in *Escherichia coli* and proofing its significant functionality leading it to produce HT. Our primary target was to investigate whether such a metabolic engineering approach could benefit the metabolic flow of tyrosine introduced to the conceived dual pathway, leading to the maximalization of the HT productivity. Various gene combinations derived from plants or bacteria were used to form a newly inspired, artificial biosynthetic dual pathway managing to redirect the carbon flow towards the production of HT directly from glucose. Various biosynthetic bottlenecks faced due to *feaB* gene function, resolved through the overexpression of a functional aldehyde reductase. Currently, we have achieved equimolar concentration of HT to tyrosine as precursor when overproduced straight from glucose, reaching the level of 1.76 mM (270.8 mg/L) analyzed by LC-HRMS. This work realizes the existing bottlenecks of the metabolic engineering process that was dependent on the utilized host strain, growth medium as well as to other factors studied in this work.

Introduction

Hydroxytyrosol (3,4-Dihydroxyphenylethanol; HT) is a mono-phenolic compound traced in olive fruits [1] and tissues [2], in extracted olive oil [3], or even in olive mills waste waters [4]. It shows a broad spectrum of biological properties due to its strong antioxidant and radical-

design, data collection and analysis, decision to publish, or preparation of the manuscript.

Competing interests: The authors have declared that no competing interests exist.

scavenging capacity [5]. Contributing most of its qualitative characteristics to olive oil, HT was recently approved as a part of "olive oil healthy polyphenols" by EC Regulation 432/2012 [6]. Concisely, the most important properties that make HT so attractive are a) its ability to scavenge the free radicals and thus acting as a potent antioxidant [7], b) the ability to reduce the risk of coronary heart disease and atherosclerosis [8, 9], c) the ability to prevent the LDL oxidation, platelet aggregation, and inhibition of 5- and 12-lipoxygenases [10], d) its critical effects on the formation and maintenance of bones, being used as an effective remedy in the treatment of osteoporosis symptoms [11] and e) its ability to control human and plant bacterial and fungal pathogens [3, 12–15].

In nature, the biosynthetic pathway for the production of HT (e.g. olives or grapes) has not been justified yet [16]. The HT content in olive fruit is highly depended on the cultivar, the cultivation techniques, the environmental conditions as well as the extraction process [17]. Owen and colleagues have comprehensively worked with the phenolic content of olives and virgin olive oil, estimating HT at about 14.4 mg/kg in oil [18, 19] and at 5.8 mg/kg at pericarp tissues [20]. In another study the HT contained in olive oil was estimated to range from 1.38 mg/kg to 7.94 mg/kg [21]. It was found that HT could be recovered from olive mill wastewaters, but low yields and complex extraction processes made recovery too expensive [22]. Interestingly, Agalias et al. [4] attempted and succeeded to purify HT from olive mill wastewaters. On the other hand, obtaining high amounts of HT by chemical synthesis is regarded cost inefficient for industrial scale production. Therefore, alternative cost-efficient strategies to produce HT at considerable high yield are desired [23].

The abovementioned properties along with the fact that it is a compound with extremely high commercial value, make its biosynthesis by biotechnological means attractive. Bioconversion of tyrosol to HT has been reported earlier by Espin et al. [24] using a mushroom tyrosinase (TYR) as a biocatalyst and by Orenes-Pinero et al. [25] by the utilization of a phenol hydroxylase gene from *Geobacillus thermoglucosidasius*. The disadvantages of this approach include the high cost and instability of the TYR enzyme. Later, a soil bacterium identified as *Pseudomonas aeruginosa*, was isolated based on its ability to grow on tyrosol as a sole source of carbon and energy [26]. During growth on tyrosol, this strain promoted the formation of HT and trace quantities of hydroxyphenylacetic acid and 3,4-dihydroxyphenyl acetic acid. Although these methods were successful for the production of HT, they were either cost-inefficient because of the required protocols for enzyme production [24] or required the supplementation of relatively expensive intermediates [26]. On the other hand, Satoh et al. [27] managed to produce HT utilizing an *Escherichia coli* system, achieving however very low titers.

Previous research attempts to produce HT directly from glucose resulted in 12.3 mg/L (0.08 mM) from *E. coli* grown in M9 broth supplemented with yeast extract [27]. They reconstituted the HT pathway utilizing a tyrosine hydroxylase, a biopterin regeneration pathway, a L-3,4-dihydroxyphenylalanine (DOPA) decarboxylase (DDC) and a tyramine oxidase expressed into JW1380 *E. coli* hosts. However recently, Chung et al. [28] followed an alternative approach by utilizing a phenyl acetaldehyde synthase to convert tyrosine into 4-hydroxyphenyl acetaldehyde, which was sequentially converted to tyrosol by an alcohol dehydrogenase (alternate name for aldehyde reductase) and finally to HT by the action of and 4-hydroxyphenylacetate 3-hydroxylase (HpaBC). This approach led to the production of HT directly from glucose at a concentration of 208 mg/L.

In this research work, we describe a strategy for the grafting of an engineered pathway into *E. coli* cells to produce HT through a dual invented pathway (Fig 1). We present a dual approach to produce HT directly from glucose, managing to score one of the highest HT concentrations produced so far. Specifically, we have tailored the so-called HT pathway with

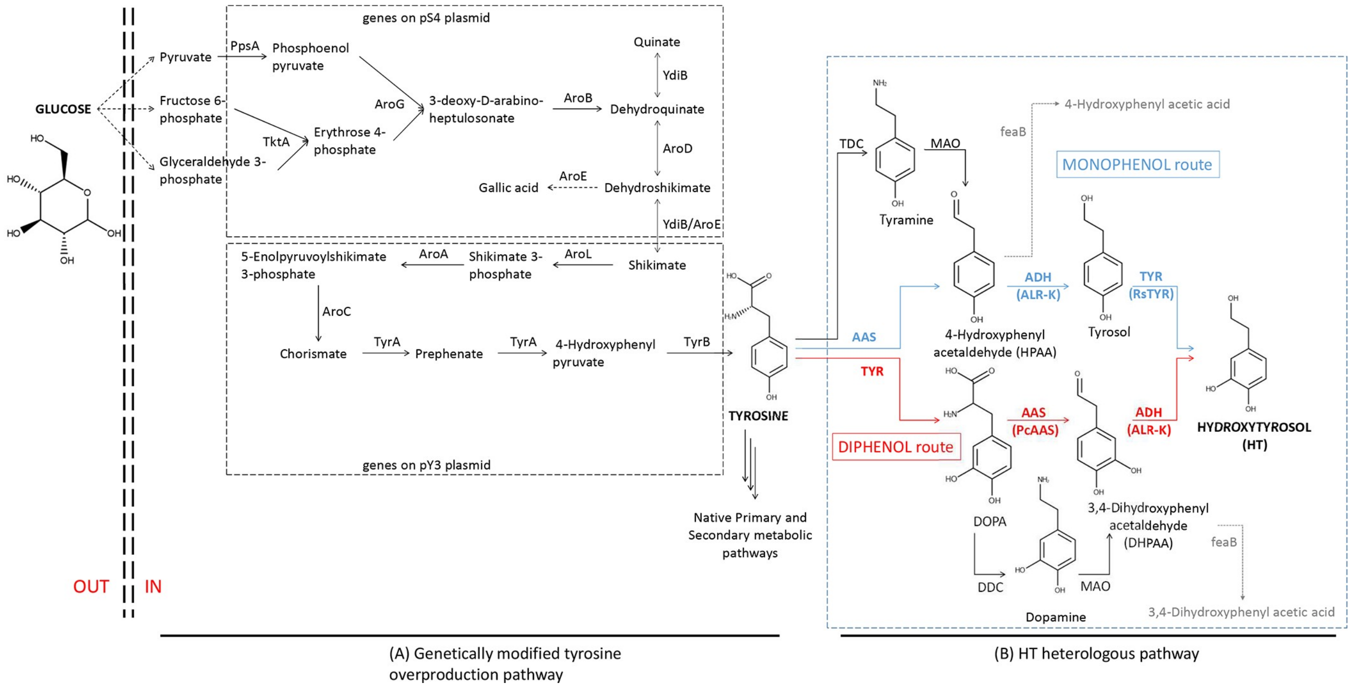

Fig 1. Metabolic scheme to produce hydroxytyrosol in *Escherichia coli* directly from glucose. (A) Tyrosine is produced through an overproducing metabolically modified machinery of the primary metabolism (Juminaga et al. 2012) and serves as the main precursor channeled to (B) the newly introduced hydroxytyrosol (HT) pathway able to transform tyrosine into HT. Double-dashed lines depict *E. coli*'s outer membrane. Dotted arrows depict multiple reaction steps. Due to the dual specificity of the utilized aromatic acetaldehyde synthase (AAS) cloned from parsley, and to tyrosinase (TYR), cloned from *Ralstonia solanacearum*, a dual pathway is generated. The "monophenol route" (blue) pathway portrays the enzymatic route for HT biosynthesis deriving from the decarboxylation-deamination of tyrosine, while the "diphenol route" (red) pathway portrays the HT route deriving from the hydroxylation of tyrosine to L-3,4-dihydroxyphenylalanine (DOPA). Gene-respective enzyme abbreviations: *AAS*, aromatic acetaldehyde synthase. *ALR*, aldehyde reductase. *AroA*, 5-enolpyruvoylshikimate 3-phosphate synthase. *AroB*, dehydroquinate synthase. *AroC*, chorismate synthase. *AroD*, dehydroquinate dehydratase. *AroE*, shikimate dehydrogenase. *AroG*, 3-deoxy-D-arabino-heptulosonate synthase. *AroL*, shikimate kinase. *DDC*, DOPA decarboxylase. *feaB*, phenylacetaldehyde dehydrogenase, *MAO*, monoamine oxidase. *PpsA*, phosphoenolpyruvate synthase. *TDC*, tyrosine decarboxylase. *TktA*, transketolase A. *TYR*, tyrosinase. *TyrA*, chorismate mutase/prephenate dehydrogenase. *TyrB*, tyrosine aminotransferase. *YdiB*, quinate/shikimate dehydrogenase (see further details in Table 1). Bold abbreviations denote the genes that were utilized in this study.

https://doi.org/10.1371/journal.pone.0212243.g001

tyrosine as a precursor, utilizing the following genes: an aromatic acetaldehyde synthase (*AAS*), an aldehyde reductase (*ALR*) and a tyrosinase (*TYR*) (Table 1). Various efforts were undertaken to optimize this heterologous HT production. We marked each factor's specific effect on the metabolic flux and on the final HT yield. To achieve this, we engineered regular strains [BL21(DE3) and HMS174(DE3)], expressing genes not only to produce HT, but also for the endogenous tyrosine overproduction, thus leading to highest titers of HT directly from glucose. Such a metabolic effect, permitted the redirection of primary metabolic flux from externally added glucose to a tyrosine pool that proved to be saturating the first enzymatic step (Fig 1) of the engineered pathway from tyrosine to HT. Moreover, the addition of a selected *ALR* (*ALR-K*, Table 1), overexpressed along with the other abovementioned genes, (Fig 1B), led to additional increase in HT production efficiency.

Materials and methods

Materials and DNA extraction

All chemicals used were purchased from Sigma-Aldrich unless otherwise stated. Genomic DNA extractions were accomplished from overnight cultures with Qiagen Blood & Tissue kit according to manufacturer instructions. PCR amplified fragments and digested fragments

Table 1. All different gene/enzyme descriptions used in this study.

Gene Abbreviation	Respective Enzyme name or Class	EC Number	Source [Ref]	Substrate specificity	Desirable product
**(A) Genetically modified tyrosine overproduction pathway (as shown in Fig 1A)**					
AroA	5-Enolpyruvoylshikimate 3-phosphate synthase	2.5.1.19	*Escherichia coli* [29]	shikimate-3-phosphate	5-enolpyruvoylshikimate 3-phosphate
AroB	Dehydroquinate synthase	4.2.3.4	*E. coli* [29]	3-deoxy-D-arabino-heptulosonate	dehydroquinate
AroC	Chorismate synthase	4.2.1.10	*E. coli* [29]	5-enolpyruvoylshikimate 3-phosphate	chorismate
AroD	Dehydroquinate dehydratase	1.1.1.25	*E. coli* [29]	dehydroquinate	dehydroshikimate
AroE	Shikimate dehydrogenase	2.5.1.19	*E. coli* [29]	dehydroshikimate	shikimate
AroG	3-Deoxy-D-arabino-heptulosonate synthase	2.5.1.54	*E. coli* [29]	phosphoenol pyruvate and erythrose 4-phosphate	3-deoxy-D-arabino-heptulosonate
AroL	Shikimate kinase	2.7.1.71	*E. coli* [29]	shikimate	shikimate 3-phosphate
PpsA	Phosphoenolpyruvate synthase	2.7.9.2	*E. coli* [29]	pyruvate	phosphoenol pyruvate
TktA	Transketolase A	2.2.1.1	*E. coli* [29]	fructose 6-phosphate and glyceraldehyde 3-phosphate	erythrose 4-phosphate
TyrA	Chorismate mutase/prephenate dehydrogenase	1.3.1.12	*E. coli* [29]	chorismate, prephenate	prephenate, 4-Hydroxyphenyl pyruvate
TyrB	Tyrosine aminotransferase	2.6.1.5	*E. coli* [29]	4-hydroxyphenyl pyruvate	tyrosine
YdiB	Quinate/shikimate dehydrogenase	1.1.1.282	*E. coli* [29]	dehydroshikimate	shikimate
**(B) HT heterologous pathway (as shown in Fig 1B)**					
ALR-K (previously named yahK [30]*)*	Aldehyde reductase (ALR)	1.1.1.2	*E. coli* [30]	hydroxyphenyl acetaldehyde	tyrosol
				dihydroxyphenyl acetaldehyde	hydroxytyrosol
ALR-D (previously named yqhD [30]*)*		1.1.1.21	*E. coli* [30]	hydroxyphenyl acetaldehyde	tyrosol
				dihydroxyphenyl acetaldehyde	hydroxytyrosol
AAS / PcAAS (previously named TDC / PcTDC [31]*)*	Aromatic Acetaldehyde Synthase (AAS)		*Petroselinum crispum* [31]	L-tyrosine	4-hydroxyphenyl acetaldehyde (4-HPA)
				L-DOPA (L-3,4-dihydroxyphenylalanine)	3,4-dihydroxyphenyl acetaldehyde (3,4-DHPAA)
TYR / RsTYR	Tyrosinase	1.14.18.1	*Ralstonia solanacearum* [32]	L-tyrosine	L-DOPA
				tyrosol	hydroxytyrosol

Enzyme protein classes used throughout the text along with the source of the gene and main biochemical information. EC numbers were extracted from BRENDA database [33].

https://doi.org/10.1371/journal.pone.0212243.t001

were purified with Macherey-Nagel NucleoSpin Gel and PCR Clean-up kit. Restriction enzymes were purchased from Minotech, Crete, Greece or New England Biolabs. Taq DNA polymerase was provided from Kapa Biotech while the proof-reading polymerase used for all the cloning processes (Phusion) was obtained from New England Biolabs. For SDS PAGE the PiNK prestained protein ladder was used (Nippon Genetics).

Genetic material, microbial hosts and cloning vectors

All gene descriptions are listed in Table 1. The *TYR* gene was cloned from the strain GMI1000 of *Ralstonia solanacearum* [32], the AAS gene was cloned from root cDNA of *Petroselinum crispum* (parsley). The *yahK* and *yqhD* genes that encode for aldehyde reductases were cloned from *E. coli* genomic DNA [30]. For further convenience, we will refer to these two genes with the abbreviations *ALR-K* and *ALR-D* respectively (Table 1). All genes involved in the tyrosine overproduction pathway (Fig 1A) were kindly donated by Prof. J. D. Keasling [29].

All cloning and sub-cloning steps were done using the Novagen pRSF and pET dual expression (Duet) vectors, all comprising the pET protein expression system. It was developed for the cloning and expression of recombinant proteins in *E. coli*. Target genes were cloned into

Table 2. Metabolically engineered bacterial plasmids (see Table 1 for gene details) and strains constructed or used in this work.

Plasmid name	Genotype
pRSF-TYR	RSF *ori kan lacI T7prom-RsTYR-T7term*
pRSF-AAS	RSF *ori kan lacI T7prom-PcAAS-T7term*
pRSF-TYR-AAS	RSF *ori kan lacI T7prom-PcAAS-T7prom-RsTYR-T7term*
pCDF-ALR-K	CDF *ori aadA lacI T7prom-yahK-T7term*
pCDF-ALR-D	CDF *ori aadA lacI T7prom-yqhD-T7term*
pS4	BBR1 *ori cat* $P_{lac\text{-}UV5}$*-aroE-aroD-aroB*^{OPT} *PLtetO-1-aroG*-ppsA-tktA dbl term* [29]
pY3	p15a *ori bla* $P_{lac\text{-}UV5}$*-tyrB-tyrA*-aroC T1 term Ptrc-aroA-aroL dbl term* [29]
**Strain name**	**Genotype**
DH10B	F- endA1 recA1 galE15 galK16 nupG rpsL ΔlacX74 Φ80lacZΔM15 araD139 Δ(ara,leu)7697 mcrA Δ(mrr-hsdRMS-mcrBC) λ-
BL21(DE3) [subsequently referred to as BL21]	F^- *ompT gal dcm lon hsd*S_B(r_B^- m_B^-) λ(DE3 [*lac*I $_{lac\text{-}UV5}$-T7 gene 1 *ind*1 *sam7 nin*5])
HMS174(DE3) [subsequently referred to as HMS174]	F^- *recA1 hsdR*(r_{K12}^- m_{K12}^+) (DE3) (Rif^R)
BL21-pRSF-TYR	BL21 RSF *ori kan lacI T7prom-RsTYR-T7term*
HMS174-pRSF-TYR	HMS174 RSF *ori kan lacI T7prom-RsTYR-T7term*
BL21-pRSF-AAS	BL21 RSF *ori kan lacI T7prom-PcAAS-T7term*
HMS174-pRSF-AAS	HMS174 RSF *ori kan lacI T7prom-PcAAS-T7term*
BL21-pRSF-TYR-AAS	BL21 RSF *ori kan lacI T7prom-PcAAS-T7prom-RsTYR-T7term*
BL21- Tyr^{OP}-HT^{OP}	BL21 [BBR1 *ori cat* $P_{lac\text{-}UV5}$*-aroE-aroD-aroB*^{OPT} *PLtetO-1-aroG*-ppsA-tktA dbl term*] [p15a *ori bla* $P_{lac\text{-}UV5}$*-tyrB-tyrA*-aroC T1 term Ptrc-aroA-aroL dbl term*] [RSF *ori kan lacI T7prom-PcAAS-T7prom-RsTYR-T7term*]
HMS174-Tyr^{OP}-HT^{OP}	HMS174 [BBR1 *ori cat* $P_{lac\text{-}UV5}$*-aroE-aroD-aroB*^{OPT} *PLtetO-1-aroG*-ppsA-tktA dbl term*] [p15a *ori bla* $P_{lac\text{-}UV5}$*-tyrB-tyrA*-aroC T1 term Ptrc-aroA-aroL dbl term*] [RSF *ori kan lacI T7prom-PcAAS-T7prom-RsTYR-T7term*]
BL21-Tyr^{OP}-HT^{OP}-ALR-K	BL21 [BBR1 *ori cat* $P_{lac\text{-}UV5}$*-aroE-aroD-aroB*^{OPT} *PLtetO-1-aroG*-ppsA-tktA dbl term*] [p15a *ori bla* $P_{lac\text{-}UV5}$*-tyrB-tyrA*-aroC T1 term Ptrc-aroA-aroL dbl term*] [RSF *ori kan lacI T7prom-PcAAS-T7prom-RsTYR-T7term*] [CDF *ori aadA lacI T7prom-yahK-T7term*]
HMS174- Tyr^{OP}-HT^{OP}-ALR-K	HMS174 [BBR1 *ori cat* $P_{lac\text{-}UV5}$*-aroE-aroD-aroB*^{OPT} *PLtetO-1-aroG*-ppsA-tktA dbl term*] [p15a *ori bla* $P_{lac\text{-}UV5}$*-tyrB-tyrA*-aroC T1 term Ptrc-aroA-aroL dbl term*] [RSF *ori kan lacI T7prom-PcAAS-T7prom-RsTYR-T7term*] [CDF *ori aadA lacI T7prom-yahK-T7term*]
BL21-Tyr^{OP}-HT^{OP}-ALR-D	BL21 [BBR1 *ori cat* $P_{lac\text{-}UV5}$*-aroE-aroD-aroB*^{OPT} *PLtetO-1-aroG*-ppsA-tktA dbl term*] [p15a *ori bla* $P_{lac\text{-}UV5}$*-tyrB-tyrA*-aroC T1 term Ptrc-aroA-aroL dbl term*] [RSF *ori kan lacI T7prom-PcAAS-T7prom-RsTYR-T7term*] [CDF *ori aadA lacI T7prom-yqhD-T7term*]
HMS174- Tyr^{OP}-HT^{OP}-ALR-D	HMS174 [BBR1 *ori cat* $P_{lac\text{-}UV5}$*-aroE-aroD-aroB*^{OPT} *PLtetO-1-aroG*-ppsA-tktA dbl term*] [p15a *ori bla* $P_{lac\text{-}UV5}$*-tyrB-tyrA*-aroC T1 term Ptrc-aroA-aroL dbl term*] [RSF *ori kan lacI T7prom-PcAAS-T7prom-RsTYR-T7term*] [CDF *ori aadA lacI T7prom-yqhD-T7term*]

The asterisks in *aroG** and *tyrA** refer to the feedback-resistant variants of *aroG* and *tyrA*, respectively [29]. OP designation stands either for a tyrosine (Fig 1A) or a hydroxytyrosol (HT, Fig 1B) overproducer strain. OPT designation stands for codon optimized version.

https://doi.org/10.1371/journal.pone.0212243.t002

pET plasmids under the control of a strong bacteriophage T7 transcription and (optionally) translation signals.

The *Escherichia coli* strain used for accomplishing the cloning strategy was the DH10B while the host bacterial strains used for expression experiments were the BL21 and HMS174 (Table 2). BL21 and HMS174 were evaluated for use as hosts because the former belongs to the B line of *E. coli*, while the second belongs to the K12 *E. coli* line [34].

Software tools

The prediction of twin-arginine translocation signal peptide in *R. solanacearum* tyrosinase was assessed with the PRED-TAT web integrated tool (ww.compgen.org/tools/PRED-TAT/submit) utilizing Hidden Markov Models [35]. Primer design and *in-silico* DNA manipulations were completed with SnapGene (http://www.snapgene.com/).

Engineering of expression vectors with genes involved in hydroxytyrosol biosynthesis

Construction of tyrosinase and aromatic acetaldehyde synthase expression vectors. All engineered plasmids constructed in this study were based on Duet expression vectors. The construction strategy and steps for each engineered plasmid used in this study are depicted in Fig 2. Genes were either cloned by PCR utilizing appropriately designed oligonucleotides from

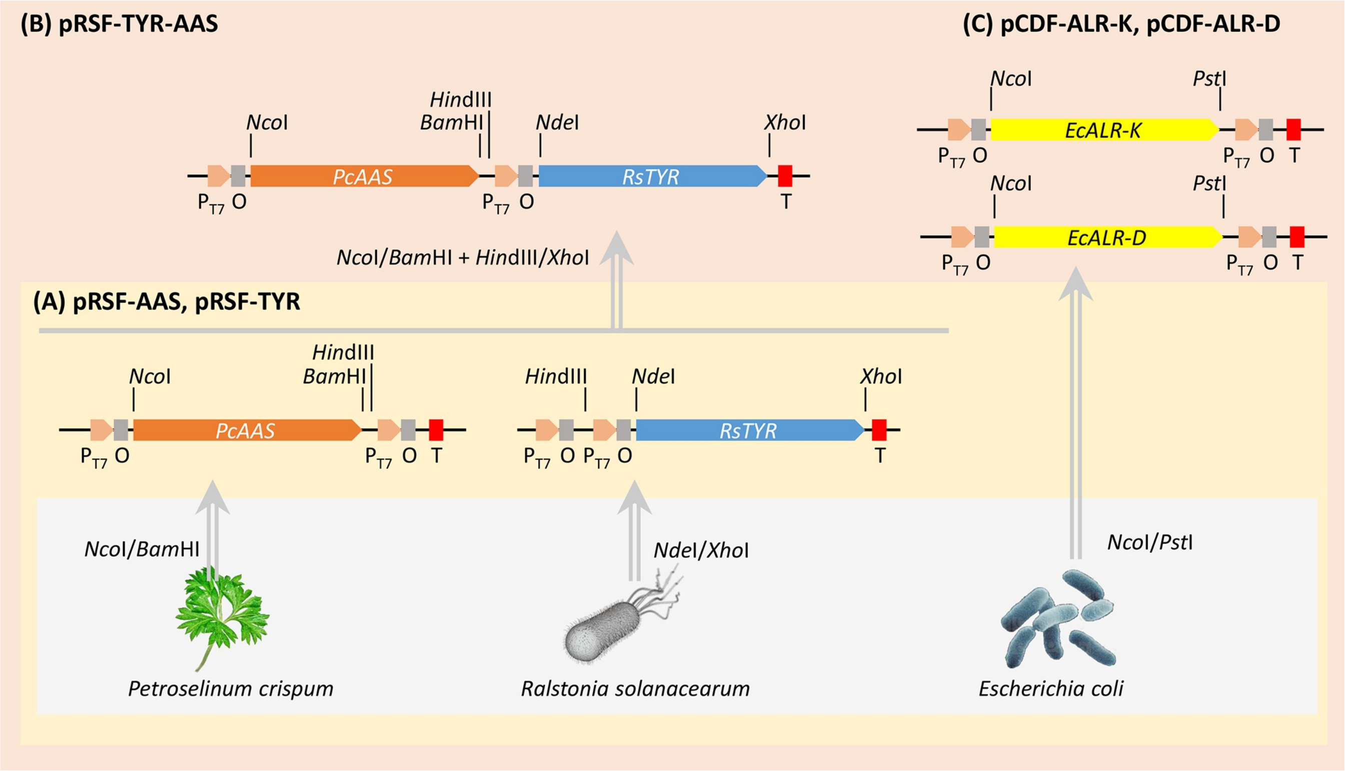

Fig 2. Schematical representation of the cloning strategy to construct the DNA plasmids used throughout this work. Three levels of constructions were created (A), (B), (C). Level (A) deals with the DNA products generated either by PCR using the appropriate primers or by direct synthesis of codon optimized (for *Escherichia coli* expression) DNA of the depicted organism. Level (B) consists of single modules into Dual expression vectors (Duet). Level (C) contains the dual gene expression vectors. Non-arrowed lines indicate the appropriately named restriction sites. Letter symbols correspond to suitable sequences: P_{T7}, T7 Promoter; O, Lac Operator; T, Termination, E.c., *E. coli*.

https://doi.org/10.1371/journal.pone.0212243.g002

various sources (S1 Table). To confirm each gene's identity the PCR cloned fragments were sequenced and compared *in silico* with the NCBI deposited sequences.

All plasmid constructions used in this study are shown in Table 2 and Fig 2. The *TYR* gene was amplified from *R*. *solanacearum* genomic DNA with the primer pair RsTYRfw2/RsTYRrv2 (S1 Table). The amplified DNA fragment was digested with the restriction enzymes *Nde*I and *Xho*I that flank the fragment and was inserted into the respective sites of an empty pRSF Duet vector (Novagen). The resulted vector was named pRSF-RsTYR. The *AAS* gene from *P*. *crispum* (parsley) was amplified from parsley root cDNA, where pathogen responsive genes [36] are usually active [37], with the primer pair PcTDC2bfw/PcTDC2bfw2 (S1 Table). The amplified DNA fragment was digested with the restriction enzymes *Nco*I and *Bam*HI and inserted into the respective site of the pRSF Duet vector generating the pRSF-PcAAS expression vector. To create the double gene expressing pRSF vector, the pRSF-RsTYR plasmid was digested with *Hin*dIII/*Xho*I enzymes to excise the RsTYR expression module and inserted into the similarly digested pRSF-PcAAS, thus creating the pRSF-PcAAS-RsTYR dual expression vector.

Construction of aldehyde reductase expression vectors. Two aldehyde reductase (ALR) genes from *E*. *coli* BL21(DE3) (*ALR-D*, and ALR-K) were cloned into pRSF Duet and pCDF Duet vectors. The *ALR-D* and ALR-K were amplified with the yqhD-fw1/yqhD-rv1 and *yahK*-fw1/*yahK*-rv1 primer pairs respectively (S1 Table) and were cloned into pCDF Duet vectors after digestion of both PCR and vector with *Nco*I/*Pst*I. The resulted plasmids were named pCDF-ALR-D or pCDF-ALR-K respectively (S1 Table).

In-vivo expression and growth optimization experiments

All genes referred in Table 1 were used primarily in *in-vivo* experiments, to check their optimal expression as well as their function. The latter was achieved by measuring their activity of their respective enzyme product activity through the quantity of their equivalent end-product biosynthesis.

In feeding experiments the LB-M9 protocol was used according to which a 5 mL starter culture was set in LB (1% tryptone, 0.5% yeast extract, 1% NaCl) from freshly transformed *E*. *coli* with appropriate plasmids and left to grow overnight at 37˚C. The next day, 50 mL of LB were inoculated with the overnight culture to OD_{600} = 0.05–0.1 and were left to grow to an OD_{600} = 0.4–0.6. At that point isopropyl β-D-1-thiogalactopyranoside (IPTG) was added at 1 mM concentration to induce the expression of the heterologous gene(s). After an induction time of 3 hours the cells were harvested by centrifugation (6000 g, 10 min), suspended in M9 medium (22.3 mM $Na_2HPO4{\cdot}7H_2O$, 22 mM KH_2PO_4, 8.6 mM NaCl, 18.7 mM NH_4Cl, 1 mM $MgSO_4$, 25 μM $CuSO_4$, 0.1 mM $CaCl_2$, 10 nM thiamine, 55 mM glucose) and appropriate substrate was added. 50 mL fermentations were performed in 250 mL Erlenmeyer flasks with orbital shaking at 200 rpm and 30˚C. For the analysis of the substrate consumption and the product biosynthesis, 1 mL samples were collected and kept at -20˚C until their electro-chromatographic analysis.

For HT production directly from glucose the simple M9 protocol was followed according to which, similarly to LB-M9, a 5 mL starter culture was set in LB with appropriate plasmids and left to grow overnight at 37˚C. The next day, 50 mL of LB were inoculated with the overnight culture to OD_{600} = 0.05–0.1 and were left to grow to an OD_{600} = 0.4–0.6. The cells were harvested by centrifugation (6000 g, 10 min), washed with M9 broth containing the basic salts and resuspended in M9 broth as in LB-M9 protocol. For the protein induction, IPTG was added at 50μM concentration. 50 mL fermentations were performed in 250 mL Erlenmeyer flasks with orbital shaking at 200 rpm and 30˚C. For the analysis, 1 mL samples were collected and kept at -20˚C until their electro-chromatographic analysis.

Tyrosinase and aromatic acetaldehyde synthase *in-vitro* enzymatic determination

To obtain bacterial cellular extracts enriched in the expressed proteins, freshly transformed BL21 clones with the appropriate expression vector were grown at 37˚C overnight in 5 mL LB. The next day 50 mL of LB in a 250 mL Erlenmeyer flask were inoculated with 1 mL of the overnight culture and left to grow for 3 hours to reach and OD_{600} of about 0.5. At that point the inducer IPTG was added to a final concentration of 1 mM. Cells were left to grow for 20 hours and then the cells were harvested by centrifugation (3000 g, 10 min, 4˚C) and washed with 50 mL of cold 20 mM Tris·HCl pH 7, harvested again and resuspended in 5 mL of the same buffer. The cells were disrupted by sonication with a Braun Labsonic U sonicator (5-min treatment at a relative output power of 0.5 with 0.5 duty period). The homogenate was centrifuged at 20000g for 30 min, and the supernatants were used for enzymatic activity determinations.

TYR activity was determined as previously described [38] for tyrosine hydroxylase. Briefly, TYR activity was carried out at room temperature in an assay (1 ml) containing 0.05% SDS, 20 mM phosphoric buffer pH 5, 1 mM tyrosine, 25 μM $CuSO_4$. The reaction was initiated upon the addition of a crude extract containing 500 μg of total proteins (described above) estimated with the Bradford assay [39]. Successive samplings of 100 μl at 0, 10, 20, 30, and 60 minutes after reaction start were analyzed in CE.

Electro-chromatographic analysis

Qualitative and quantitative analysis of samples were carried out in an Agilent Capillary Electrophoresis (CE) G1605 system coupled with a Diode Array Detector (DAD). A bare fused silica capillary column was used with effective length of 50 cm and inner diameter of 75 μm. The analysis was carried out in several steps: preconditioning, inlet and outlet buffer replenishment, buffer customization, injection and analysis. The preconditioning step involved an initial flush with 1 N sodium hydroxide for 2 min followed by a flush with analysis buffer (25 mM sodium tetraborate decahydrate, pH 9.2) for 4 min. Buffer customization included the application of 20 kV voltage for 2 min before each analysis The injection step comprised a 50 mbar pressure for 2 sec and the analysis was performed at 29 kV for 10 min, time sufficient for maximum resolution of the compounds analyzed in this study. The temperature of the column cassette was kept constantly at 25˚C. Replenishment and buffer customization steps were necessary to eliminate poor reproducibility of the peaks resulted from electrolytic phenomena in the running buffer and compound instability. Compounds under study were identified by matching the retention time, UV-absorbance spectrum, and co-chromatography with authentic chemicals. Calibration curves were obtained with authentic compound solutions of various concentrations.

All experiments were performed at least 3 times unless otherwise stated. Data of each experiment were used to calculate the mean and the respective standard deviations.

Mass spectrometry analysis of HT production

Further qualitative and quantitative analysis was performed on the most efficient metabolically engineered *E. coli* strain that produced the highest concentration of HT. The *E. coli* strain HMS174-TYR^{OP}-HT^{OP}-ALR-K (Table 2) was grown in 5 L flask filled with 1 L of growth medium. After 48 h of growth, samples of metabolically engineered *E. coli* strain were analyzed through mass spectrometry.

For the qualitative and quantitative monitoring of the *E. coli* strains an Accela HPLC system (Thermo), coupled to a hybrid LTQ Orbitrap Discovery XL mass spectrometer (LC–HRMS)

and equipped with an electrospray ionization source (ESI) was used. The separation was conducted using a gradient elution system consisted of water with 0.1% (v/v) formic acid (A) and acetonitrile (B). The elution started with 5% B and reached 50% in 5 min. After 2 min the system returned to the initial conditions and stayed for 5 min for equilibrium of the column. The flow rate was set at 0.4 ml/min and the total run time was 15 min. For the chromatographic separation a Fortis C-18 (1.7 μm, 150 x 2.1 mm) column was used heated at 40˚C. The injection volume was 10 μl and samples were maintained at 10˚C during analysis. Ionization was achieved in negative ion mode (ESI-) at 350˚C. The mass spectrometric parameters were: sheath gas and aux gas flow rate 40 and 10 units respectively; capillary voltage -40 V and tube lens -69 V. The mass range was adjusted from 113 to 1000 *m/z*.

In order to quantify HT, calibration curves were built using six different concentration levels of the analyte. The selected levels were 1 μg/ml, 2 μg/ml, 4 μg/ml, 6 μg/ml, 8 μg/mL and 10 μg/ml. 2.4-Dinitrophenol was used as internal standard (IS) in the concentration of 0.3 μg/ml. The construction of the calibration curve based on the ratio area of HT/IS versus the concentration of HT. Linearity was evaluated by coefficient of determination (R^2) from the linear regression using the least squares equation. All concentration levels were measured in triplicates. Blank samples were injected every three injections in order to avoid carryover effect of the different concertation levels. Data were acquired, analyzed and processed with the Thermo Xcalibur 2.1 software.

For the bacterial strain extraction, 225 mL of cell culture were centrifuged at 3500 rpm for 20 min. The supernatant was extracted with the adsorption resin Amberlite XAD7 after overnight treatment, in order to obtain an extract being enriched in phenolics. Methanol was used as extraction solvent. The obtained methanol extract was dried under vacuum until dry and reconstituted to 100 μg/mL for the HPLC-ESI-HRMS analysis.

Results

Tyrosine overproducing strain

We utilized two already constructed plasmids that were donated by Prof. Keasling's group (UC, USA), the pS4 and pY3 [29] that in total harbor 11 genes (S2 Fig) in order to overproduce tyrosine from phosphoenolpyruvic acid (PEP) and erythrose-4-phosphate (E4P) (redrawn in Fig 1A). The *E. coli* strains bearing both pS4 and pY3 behaved as tyrosine overproducers (BL21-TY^{OP} or HMS174-TYR^{OP}, Table 2). According to Juminaga et al. [29], six of these genes were necessary for the biosynthesis of shikimic acid (pS4) and five of them required for the conversion of shikimic acid into tyrosine (pY3). The shikimic acid biosynthesis module (pS4) bears the following genes under the lac-UV5 or tetO promoters: phosphoenolpyruvate synthase (*PpsA*), transketolase A (*TktA*), 3-deoxy-D-arabino-heptulosonate synthase (*AroG*), Dehydroquinate synthase (*AroB*), Dehydroquinate dehydratase (*AroD*), shikimate dehydrogenase (*AroE*). The tyrosine module (pY3) contains genes under the lac-UV5 or the trc promoters: chorismate mutase/prephenate dehydrogenase (*TyrA*), tyrosine aminotransferase (*TyrB*), chorismate synthase (*AroC*), 5-enolpyruvoylshikimate 3-phosphate synthase (*AroA*), shikimate kinase II (*AroL*). The 2 plasmids (pS4 and pY3) bearing all the necessary genes were inserted into BL21 and HMS174 *E. coli* strains (Table 2) by electroporation. The resulted tyrosine-overproducing strains, BL21-Tyr^{OP} and HMS174-Tyr^{OP}, were evaluated for their ability to produce tyrosine. The HMS174 strain presented higher ability to produce tyrosine achieving a maximal concentration of 6.23±0,18 mM.

Expression of genes involved in the biosynthesis of hydroxytyrosol

***Ralstonia solanacearum* tyrosinase.** The hydroxylation of the phenolic ring of tyrosine or tyrosol to form DOPA or HT respectively may be catalyzed by the action of the *R. solanacearum*

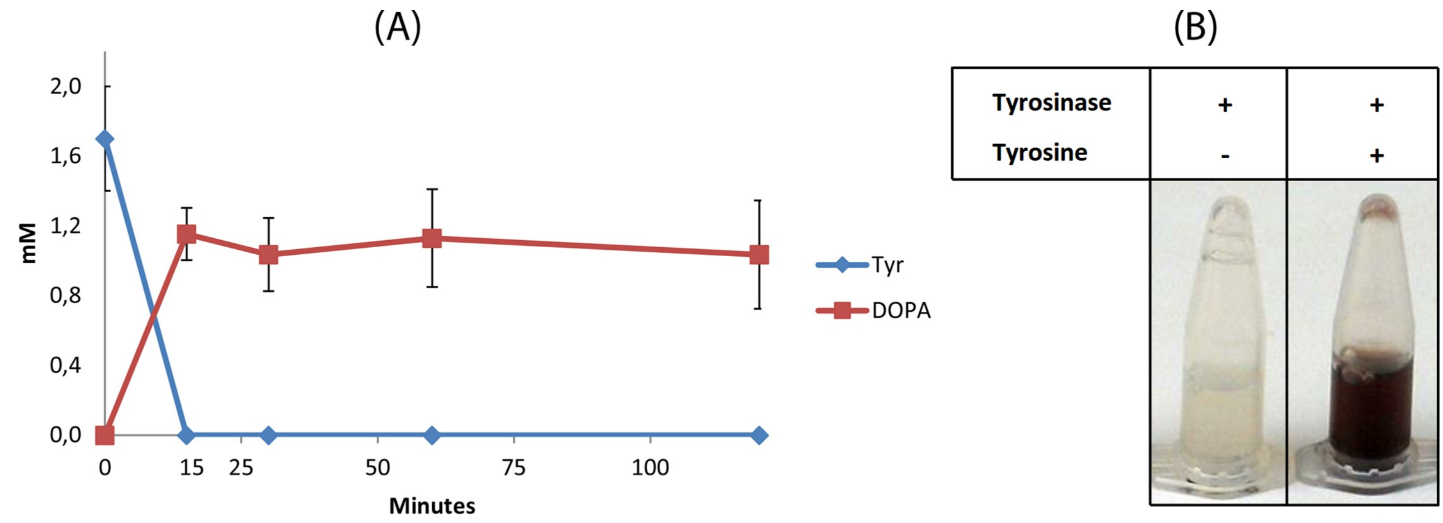

Fig 3. *In vitro* assays for the evaluation of tyrosinase activity. (A) The reaction was supplemented with 2 mM of tyrosine and DOPA production was followed. (B) Blackening of reaction due to the melanization of the produced DOPA (o-diphenolase activity of RsTYR).

https://doi.org/10.1371/journal.pone.0212243.g003

TYR (Table 1). BL21 cells bearing the RsTYR (BL21-pRSF-RsTYR, Table 2), in induced conditions were initially checked for their ability to induce the expression of the TYR protein (S3A Fig). As negative controls, BL21 cells were used transformed either with empty pRSF vector (in non-induced conditions) or with pRSF-RsTYR (also in non-induced conditions). It was obvious that an intense band (see arrow in S3A Fig), that did not exist in negative controls, was present above 43 KDa, corresponding to the TYR protein size of about 50 kDa [32]. Crude extracts from these cultures (non-induced BL21-pRSF, non-induced BL21-pRSF-RsTYR, and induced BL21-pRSF-TYR) were tested for TYR activity in a colorimetric assay since the dual activity of the enzyme oxidizes DOPA to the orange dopachrome and eventually to blackish melanins (Fig 3B). Conclusively, both the extracts from the induced and non-induced conditions were able to transform tyrosine to DOPA (and eventually to melanins) indicating from one hand the activity of the enzyme and on the other hand the leaky expression of TYR in non-induced conditions.

Subsequently, TYR activity was verified by *in-vitro* (Fig 3A) as well as colorimetric assays (Fig 3B). A time course experiment following tyrosine and DOPA certified that tyrosine was promptly converted to DOPA (Fig 3A). In all cases the color of the reaction was getting black indicating the melanization of DOPA through the diphenolase activity of TYR.

Furthermore, *in-vivo* assays for the conversion of 2 mM tyrosine or tyrosol to DOPA or HT respectively were set up (Fig 4). In induced conditions, the substrate (tyrosine or tyrosol) was converted to DOPA or HT respectively 2 h after the addition of the substrate (Fig 4B and 4D). When the cultures were kept under non-induced conditions, no consumption of the substrate was recorded, or the consumption was minimal. A slight increase of the products was also recorded (Fig 4A and 4C).

According to PRED-TAT algorithm [35] and the integrated web tools (http://www.compgen.org/tools/PRED-TAT), the N-terminal of *R. solanacearum* TYR (497 aa) was found to bear a 31 aa peptide for translocation of the protein to the periplasm [40] (S1 Fig) with reliability score of 0.998 (new model, max value 1).

***Petroselinum crispum* aromatic acetaldehyde synthase.** Beyond the hydroxylation of the phenolic ring, another essential reaction is the decarboxylation-amination of the aromatic amino acid by the respective AAS. Newly transformed BL21 cells bearing the PcAAS

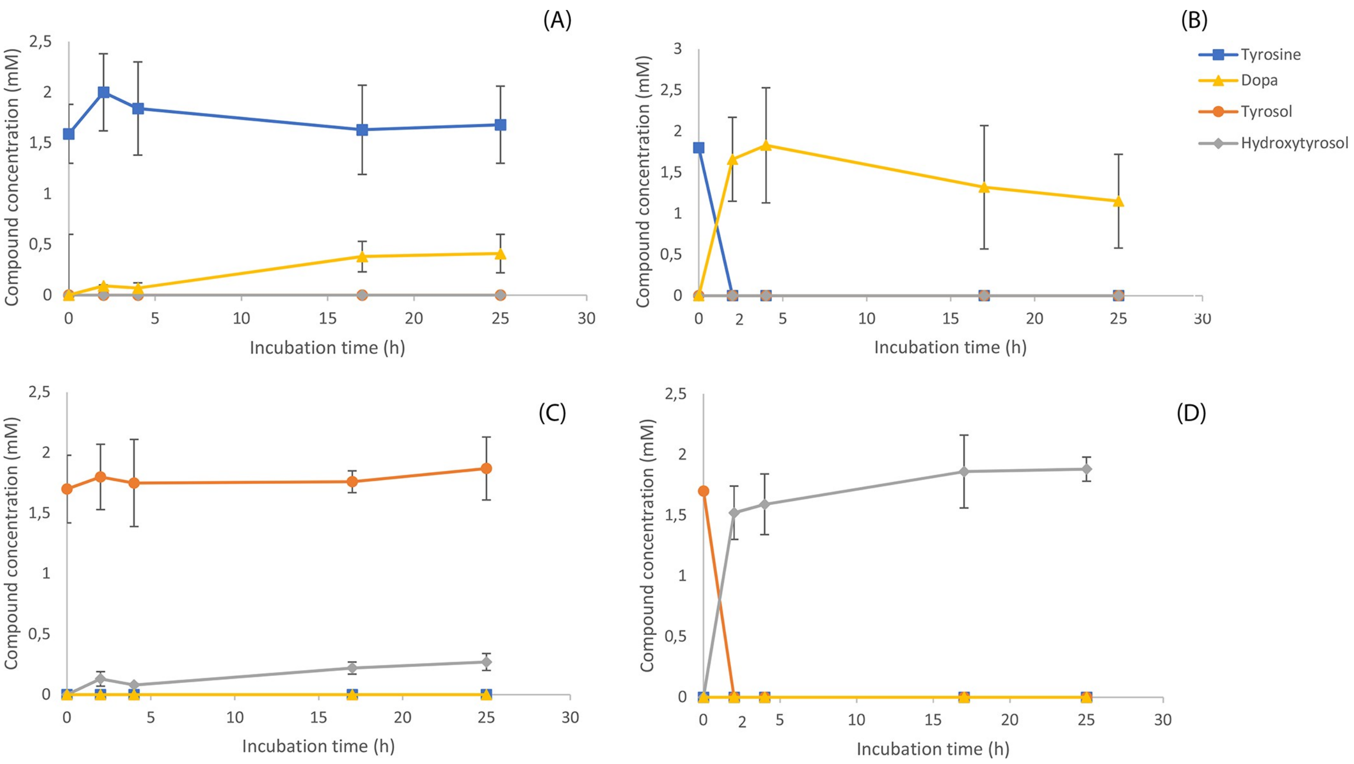

Fig 4. *In-vivo Ralstonia solanacearum* tyrosinase assay acting on supplemented tyrosine or tyrosol. *In-vivo Ralstonia solanacearum* tyrosinase assay acting on supplemented tyrosine or tyrosol. Panels A and B refer to the *in-vivo* experiment supplied with tyrosine while panels C and D refer to the experiment supplied with tyrosol. The panels at the left of the figure (A and C) depict non-induced conditions while the panels on the right part depict (B and D) the experiment from induced conditions.

https://doi.org/10.1371/journal.pone.0212243.g004

(BL21-pRSF-PcAAS, Table 2) in induced conditions were checked for their ability to induce the expression of the AAS (S3B Fig). As negative control, BL21 cells transformed with pRSF-PcAAS, in non-induced conditions, were used. As with TYR producing BL21 cells, a band with much higher intensity was present at about 50 KDa [36], corresponding to the AAS protein.

In-vivo assays for the evaluation of AAS were set up. Induced cultures were supplemented either with tyrosine or DOPA and time course samples were analyzed in CE (Fig 5). Either substrate, tyrosine or DOPA, was converted to tyrosol or HT respectively, with the latter substrate (DOPA) to be consumed at about 5 hours after substrate addition. Similarly to TYR *in-vivo* assays, the cultures under non-induced conditions presented minimal consumption of the substrate (Fig 5A and 5C) while the induced conditions exerted the maximal conversion efficiency (Fig 5B and 5D).

HT production from engineered *E. coli* strains and IPTG effect

The proof that *RsTYR* and *PcAAS* genes were active, was followed by the combined *in-vivo* expression. Cultures of BL21 cells transformed with pRSF-PcAAS-RsTYR (Table 2) were set up to evaluate their ability to convert supplemented tyrosine into HT in both induced and non-induced conditions (Fig 6A). As a rule, 1 mM of tyrosine was supplemented. The strain was evaluated on different IPTG concentrations; 0, 50, 100, 250, 500, 750, and 1000 μM. A first note was the substrate loss to melanin accumulation through the oxidation of the tyrosine derived DOPA through dopachrome observed by the blackening of the media. As the IPTG

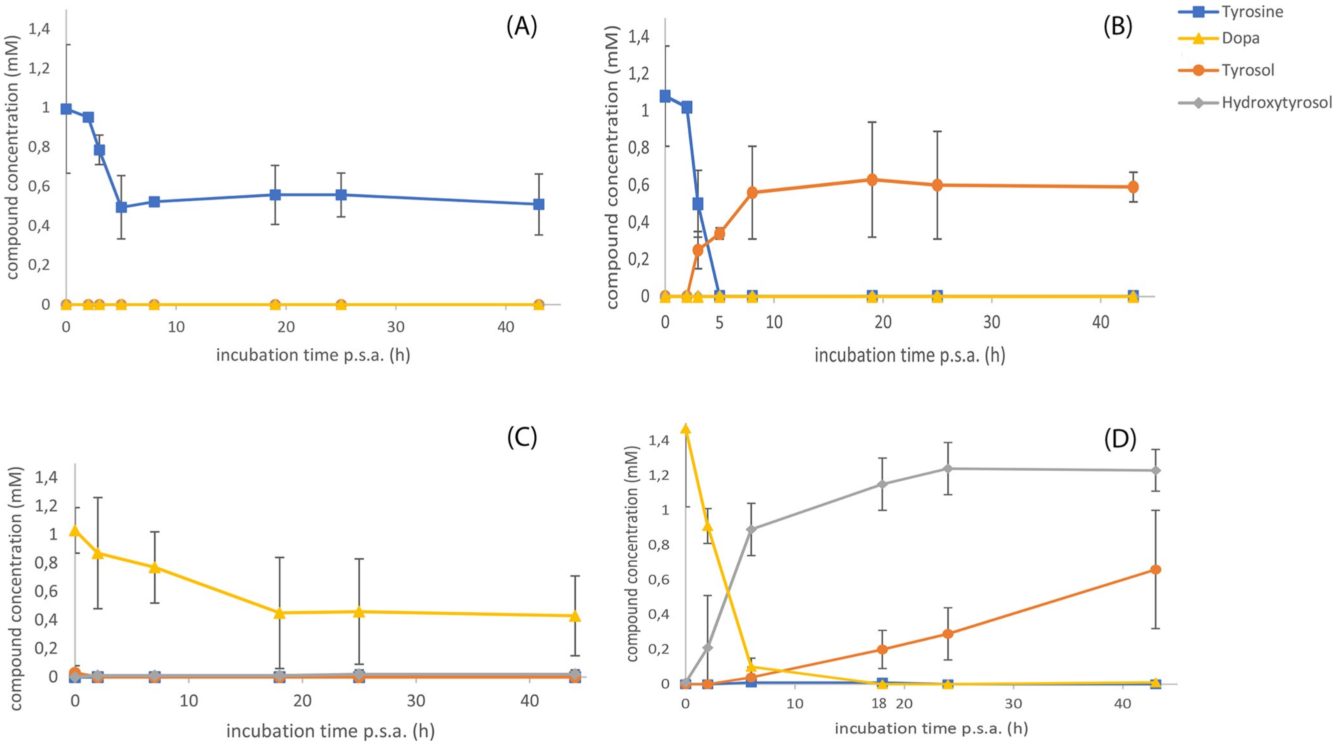

Fig 5. *In-vivo Petroselinum crispum* aromatic acetaldehyde synthase assay acting on supplemented tyrosine or DOPA. Panels A and B refer to the *in-vivo* experiment supplied with tyrosine while panels C and D refer to the experiment supplied with DOPA. The panels at the left of the figure (panels A and C) depict non-induced conditions while the panels on the right part depict (panels B and D) the experiment from induced conditions.

https://doi.org/10.1371/journal.pone.0212243.g005

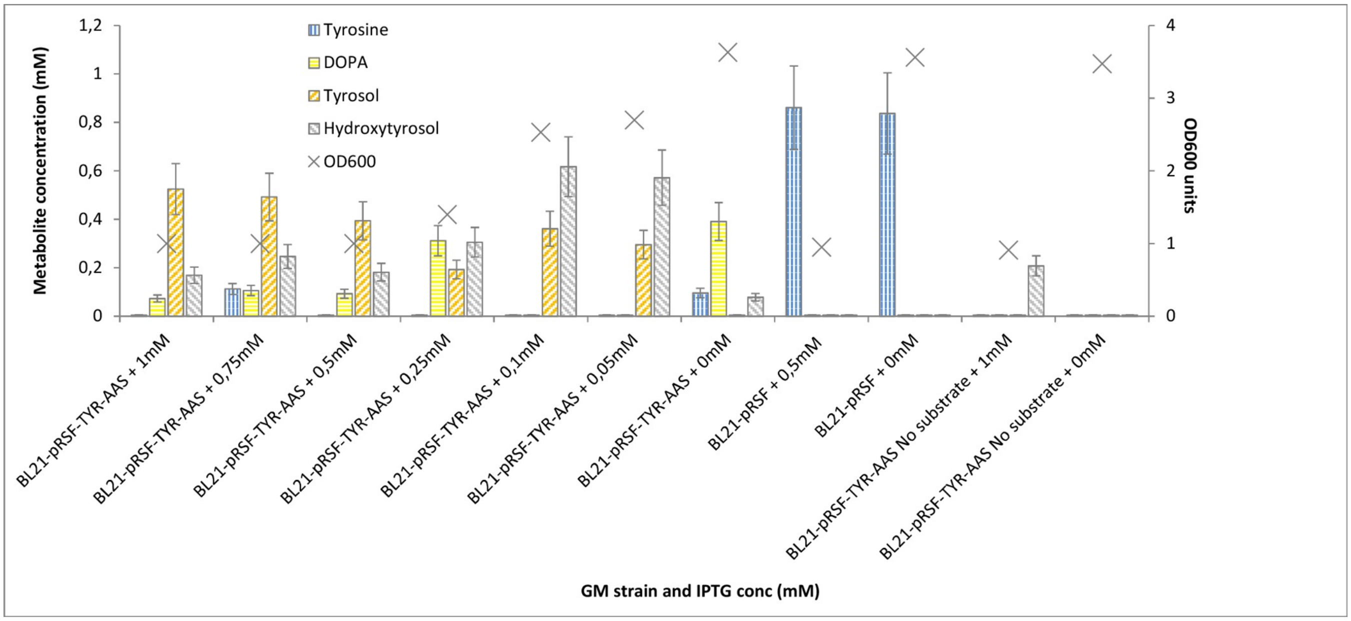

Fig 6. *In-vivo* evaluation of the BL21-TYR-AAS for its ability to produce hydroxytyrosol on various inducer concentrations. Tyrosine was initially supplemented at 1mM concentration and HT production as well as the intermediate compounds, DOPA and Tyrosol, were estimated. Values are from 48 h post substrate addition. The "X" sign estimates the cell density value.

https://doi.org/10.1371/journal.pone.0212243.g006

concentration decreased from 1 mM to 0 mM, differences were observed in the produced analytes; the intermediate tyrosol was lowering while the concentration of HT increased. Furthermore, in the lower IPTG concentrations (50 and 100 μM) DOPA was not traced. When no tyrosine was added to the system a minimal amount of HT (0,21 mM) was produced in induced conditions or no HT was produced in non-induced conditions. The consumption of tyrosine in non-induced conditions (0 mM IPTG, Fig 6) further propose the leaky activity of TYR and AAS. The negative control BL21 cells that did not carry any of the HT pathway genes, did not consume the exogenous supplied tyrosine. Consequently, 50 μM of IPTG was regarded as the optimal concentration of the inducer and was used throughout our experimental conditions unless stated otherwise.

Hydroxytyrosol production from engineered tyrosine-overproducer *E. coli* strains

Instead of using an *E. coli* system that is necessary to be supplemented with the precursor amino acids tyrosine or DOPA to produce HT, a biological system designed to overproduce tyrosine was further engineered by introducing the pS4 and pY3 plasmids to BL21 and HMS174 hosts, for the production of HT directly from glucose (BL21-TYR^{OP}-HT^{OP} and HMS174-TYR^{OP}-HT^{OP} strains, Table 2). Experiments with these strains showed non-consistent tyrosine production, while HT levels remained similar reaching the level of 0.21 mM in HMS174 strain (Fig 7). This was much an unexpected result, since there was a noticeable difference on tyrosine pool, between the two strains. While in BL21-TYR^{OP}-HT^{OP} appeared that all internally overproduced tyrosine was at nearly non-existing levels indicating that it was all consumed in primary metabolism (Fig 7A), in HMS174-TYR^{OP}-HT^{OP}, tyrosine levels reached 1.3 mM. Similar biosynthetic levels were obtained for the intermediate metabolites DOPA and tyrosol in HMS174-TYR^{OP}-HT^{OP}, however, leading to still low HT level. This metabolic event indicated that a probable biosynthetic bottleneck existed in the conversion of the internally produced tyrosine to HT.

Auxiliary aldehyde reductase genes. The biosynthetic pathway of this work utilizes the AAS from *P. crispum* (Table 1) that possess dual specificity in respect to the amino acid that is accepted as substrate. It can convert tyrosine or DOPA to 4-hydroxyphenylacetaldehyde (HPAA, monophenol route) or 3,4-dihydroxyphenylacetaldehyde (DHPAA, diphenol route) respectively (Fig 1B). The next step should be the reduction of the phenylacetaldehydes to the respective alcohols, tyrosol or HT. *E. coli* is an organism that possesses several ALRs that may drive the abovementioned conversions [27]. We therefore checked whether the presence of an extra overexpressed ALR could confer any advantage to the engineered system. The ALRs evaluated were the ALR-K and the ALR-D [30] both of which are present in the genomes of BL21 and HMS174 strains. The over-expression of *ALR-D* along with the HT biosynthesis genes did not give any advantage to the production system, either expressed in BL21 or HMS174 strain. Though, this was not the case for the over-expression of *ALR-K*. It gave a decrease of 63% in the production of HT when expressed in BL21-TYR^{OP}-HT^{OP} (Fig 8A) while it gave an increase of 386% when expressed in HMS174-TYR^{OP}-HT^{OP} (Fig 8B) reaching a concentration of 1.02 mM (157.2 mg/L) when analyzed in CE-DAD system. All the follow up experiments continued with optimization of this highest HT titer producing strain.

Optimal analysis of the highest HT producer strain through Mass Spec analysis. The strain HMS174-TYR^{OP}-HT^{OP}-ALR-K (Table 2), carrying the HT overproducing pathway (involving the genes *RsTYR*, *PcAAS* and *EcALR-K*, Table 1), as well as all the genetic accessories to overproduce tyrosine ([29], Table 1), was further analyzed by an LC-MS-MS type mass spectrometry apparatus. In this strain, the precursor of HT pathway, tyrosine (Fig 1) was

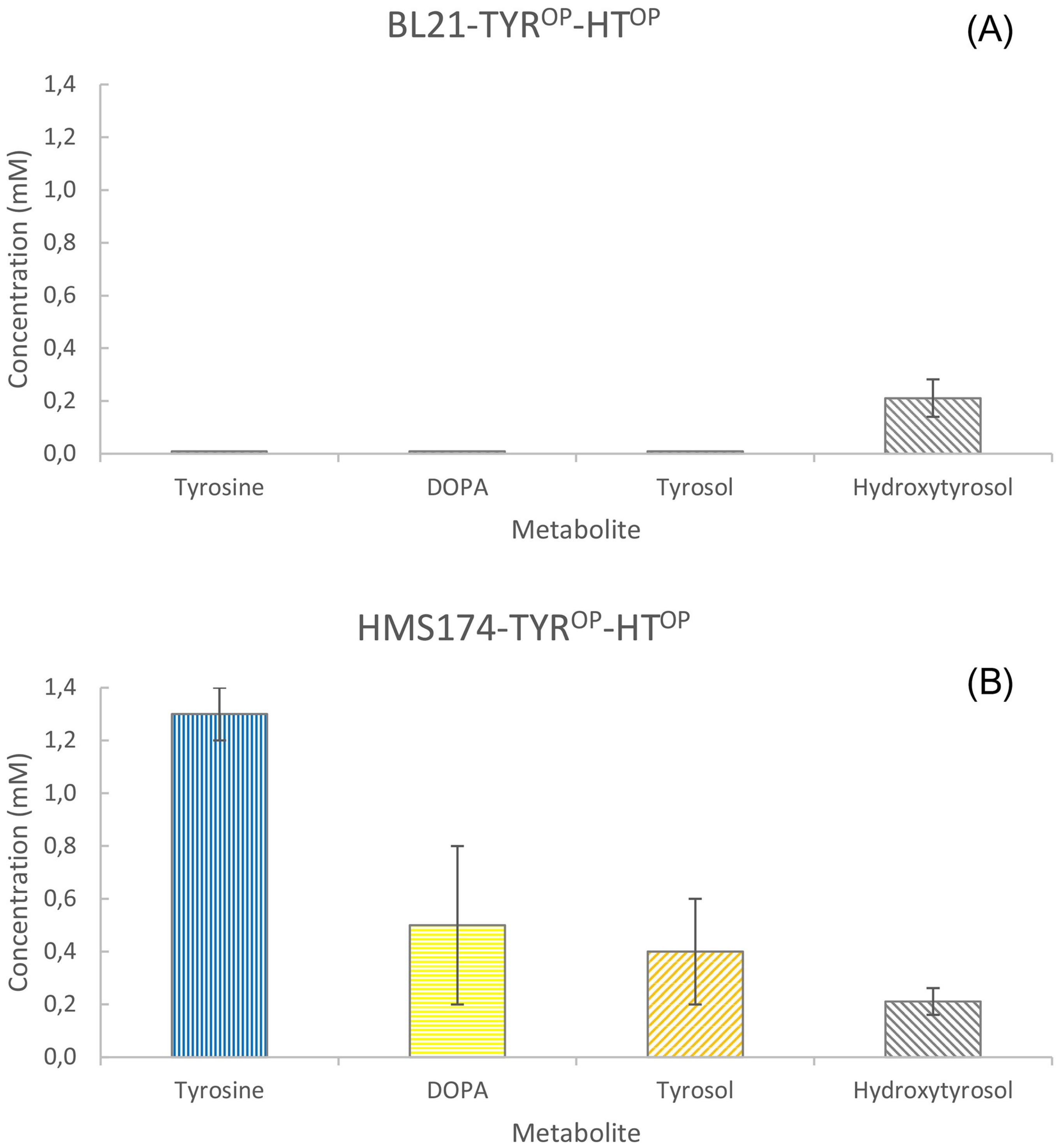

Fig 7. Hydroxytyrosol production from HMS174-TYR-^{OP}-HT^{OP} strain. Hydroxytyrosol production from BL21 (A) or HMS174 (B) *Escherichia coli* strain bearing the modules for tyrosine and hydroxytyrosol overproduction. Values are extracted from 48 h of cultivation.

https://doi.org/10.1371/journal.pone.0212243.g007

internally produced by feeding the strain with glucose. This strain grown under the M9 protocol, showed the highest HT production when samples were analyzed from 50 mL cultures (Fig 8B) with a CE-DAD system. However, it was important to find whether there was any effect

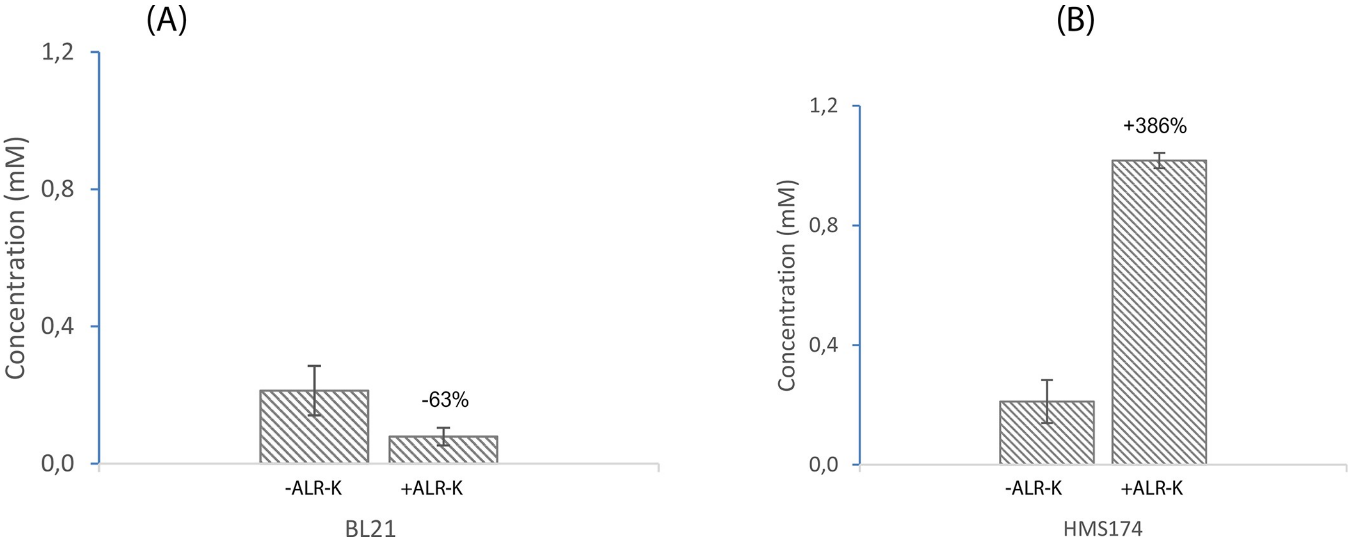

Fig 8. Evaluation of the effect of aldehyde reductase gene on the production of hydroxytyrosol. Evaluation of the effect of aldehyde reductase gene (*ALR-K*) on the production of hydroxytyrosol from BL21-TYR^{OP}-HT^{OP}-ALR-K (A) or HMS174-TYR^{OP}-HT^{OP}-ALR-K (B) strains. The values were extracted at 48 hours of cultivation. The effect of the *ALR-K* in the HT production is also presented as a percentage value (negative or positive) above the bar representing the strain engineered to express the *ALR-K*.

https://doi.org/10.1371/journal.pone.0212243.g008

on HT production when grown on large volume medium (1 L) and analyzed by mass spectroscopy. Indeed, the MS analysis showed HT production at much higher concentration level.

After the purification and enrichment process of the strain HMS174-TYR^{OP}-HT^{OP}-ALR-K using adsorption resins, the dried weigh of the methanol extract was estimated at 4.2 mg/mL

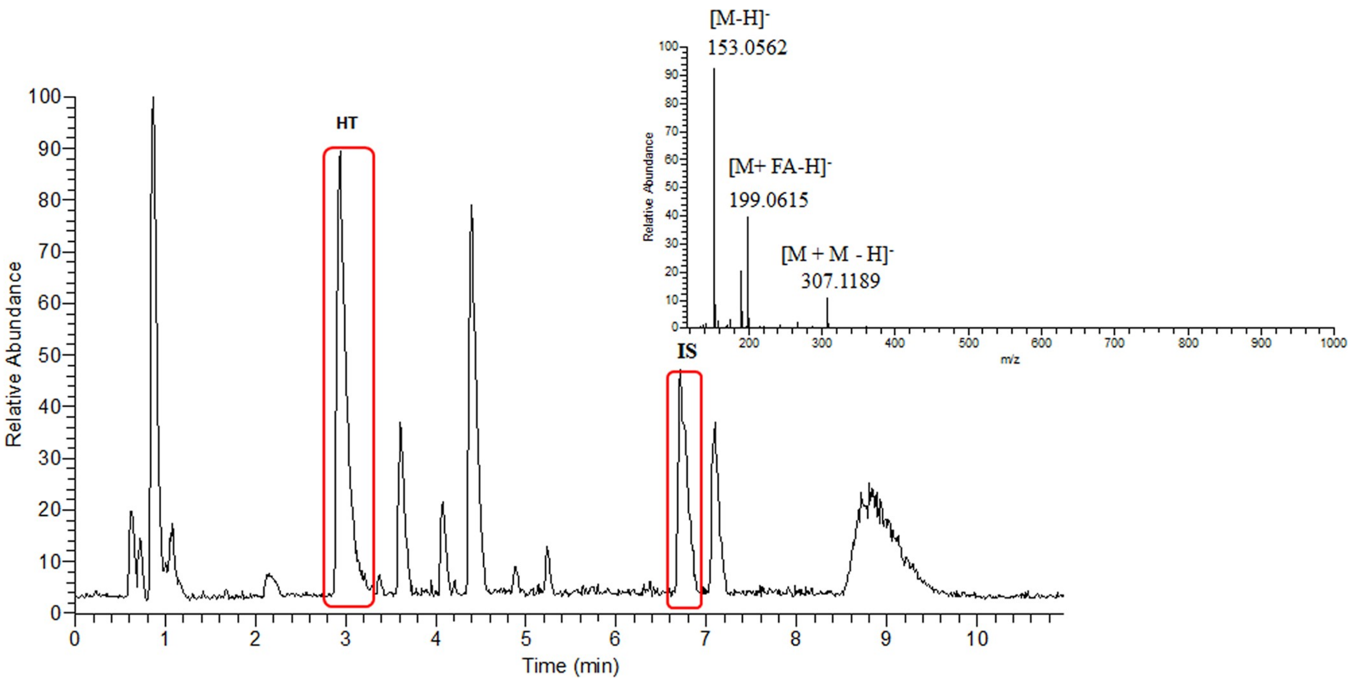

Fig 9. Base-peak HPLC-ESI-MS chromatogram of the methanol extract of the strain HMS174-TYR^{OP}-HT^{OP}-ALR-K. The two peaks circled in red are hydroxytyrosol (HT; left) and internal standard (dinitrophenol, IS, right). At the mass spectrum the characteristic ions of HT are denoted (pseudomolecular ion [M-H]- of HT at *m/z* 153.0562, the [M+FA-H]- adduct ion of HT with formic acid and the pseudodimer ion [M+M-H]- of HT), produced during the ionization process.

https://doi.org/10.1371/journal.pone.0212243.g009

cell culture. In order to determine the HT levels, HPLC-ESI(-)-HRMS was employed. Fig 9 illustrates the obtained base-peak chromatogram of the extract after resin treatment together with the corresponding mass spectrum of HT. As it is highlighted in red, HT was eluted at 2.94 min and the dinitrophenol internal standard at 6.71 min. Other peaks were also detected, mainly representing intermediate compounds of the metabolic pathway. At the mass spectrum (Fig 9, smaller graph) the ions corresponding to HT were presented. The high accuracy and resolving power of the Orbitrap analyzer as well as the use of reference standard enabled the unambiguous identification of HT in the complex mixture [41]. Together with the pseudo-molecular ion of HT ($[M-H]^-$), other ions corresponding to its adducts with formic acid ($[M+FA-H]^-$) and its dimer ($[2M-H]^-$) were also detected. Using Thermo Xcalibur 2.1 software and extraction ion method (XIC), the quantification of HT took place using the linear regression method. A standard calibration curve was built described by the equation $y = 0.1674x + 0.1929$ while coefficient of determination was calculated ($R^2 = 0.9932$), indicating the good linearity achieved. Based on the equation, the level of HT was calculated at 270.8 mg/L of cell culture.

Discussion

In this project we succeeded to modularly engineer both the primary and secondary metabolism of *E. coli* to produce HT. We chose a rational approach for its production [16] where the primary metabolism was engineered in order to boost tyrosine production that is subsequently utilized for the biosynthesis of HT through the engineered secondary metabolic machinery.

The HT molecule bears a benzene (phenolic) ring linked with an alcoholic chain with two carbon atoms (Fig 1). Since all phenolic compounds encountered in organisms emanate from the aromatic amino acids via the shikimic acid pathway [42, 43], we assumed that this is also the case for HT biosynthesis. From the three aromatic amino acids of the primary *E. coli* metabolism (phenylalanine, tyrosine, tryptophan), HT resembles most with tyrosine (Fig 1) and could be biosynthesized by a hydroxylation, a decarboxylation, a deamination, and finally a reduction (Fig 1B).

Thus, we chose to utilize the *TYR* gene from *R. solanacearum* (Table 1) for the hydroxylation of tyrosine (or tyrosol) [32] and the *AAS* gene from parsley [36]. The TYR enzyme beyond the ortho-hydroxylation of the phenolic ring also oxidizes the hydroxyl groups to quinones [24, 32]. On the other hand, the AAS enzyme has also the advantage that beyond the basal decarboxylation reaction, catalyzes an extra deamination step that leads to the production of the corresponding phenylacetaldehyde [31]. The activity of *R. solanacearum* TYR was confirmed in *in-vitro* (Fig 3) as well as in *in-vivo* experiments on tyrosine (Fig 4A and 4B) and on tyrosol (Fig 4C and 4D) while the activity of the parsley AAS was confirmed in *in-vivo* experiments where reactions were supplemented with either tyrosine (Fig 5A and 5B) or DOPA (Fig 5C and 5D). The reduction of the produced aromatic phenylacetaldehyde (4-hydroxyphenylacetaldehyde produced from the monophenol route or DHPAA produced from the diphenol route) was accomplished by internal ALRs that both BL21 and HMS174 *E. coli* strains possess [27]. The simultaneous expression of *RsTYR* and *PcAAS* was evaluated by the ability of the system to transform the supplemented tyrosine to HT (Fig 6) and proved to be a successful strategy for the robust manufacturing of the high-valuable compound HT.

The dual specificity of both utilized enzymes, TYR acting on tyrosine or tyrosol and AAS acting on tyrosine or DOPA, unwraps two possible metabolic routes for the production of HT; one through the decarboxylation-deamination of DOPA (diphenol route, Fig 1B) for the production of DHPAA, and one through the decarboxylation-deamination of tyrosine for the production of 4-hydroxyphenylacetaldehyde (monophenol route). Both parallel occurring metabolic routes, lead to the production of HT.

The analysis of RsTYR in *in-vitro* and *in-vivo* activity experiments (Fig 3 and Fig 4), where the precursor molecules were exogenously supplemented, revealed that the enzyme was active in both induced and uninduced conditions. More interestingly, it performed quickly, within the first 20 min with the *in-vitro* crude *E. coli* extract. This was advocated by the identification of DOPA in early time points upon the supplementation of tyrosine. The *in-vivo* rapid conversion of the medium supplemented tyrosine could be explained by the finding that RsTYR was transferred to the medium through the periplasmic space of the host as it possesses a TAT signal peptide (S1 Fig). The extracellularly produced DOPA was not directly available to AAS, that was intracellularly expressed. This differential compartmentalization may explain the reason why extracellular DOPA could not be easily converted to HT and was wasted as melanized byproducts due to exposure of supplemented tyrosine to RsTYR, leading to the oxidation of DOPA accompanied by the blackening of the medium (Fig 3B). Consequently, the late increase in HT is assumed to be produced through the newly synthesized endogenous tyrosine and the monophenol route (Fig 1). In that case, the intracellularly AAS-synthesized tyrosol was transferred outside of the cell before its transformation to HT by TYR.

The ability of the engineered *E. coli* to bio-transform the supplemented precursor tyrosine into the valuable HT as well as the fact that tyrosine was the limiting factor for producing HT (since its production rate was rapidly reduced to zero after tyrosine consumption; Fig 5B), urged us to evaluate the case of producing HT from a tyrosine over-producing strain. The HMS174 strain proved more efficient to produce tyrosine than BL21 as the former was able to convert the supplemented glucose to 6.23 mM of tyrosine. The efficient biosynthesis of tyrosine was necessary to avoid the exogenous supplementation of tyrosine and thus making the biological production of HT more economically attractive. To this extend, *E. coli* strains containing the modules for a) the biosynthesis of shikimic acid (pS4), b) the production of tyrosine from shikimic acid (pY3), and c) the production of HT from tyrosine, were utilized (BL21-TYR^{OP}-HT^{OP} and HMS174- TYR^{OP}-HT^{OP}) to produce HT. As was earlier mentioned, the supplementation of tyrosine to BL21-RsTYR-PcAAS or HMS174-RsTYR-PcAAS strains led to intermediate product formation due to spatial separation of the TYR and AAS activities. The channeling of intracellularly produced tyrosine to the monophenol route of HT pathway (Fig 1) resulted in the formation of tyrosol that was eventually excreted into the medium where it was converted to HT. However, both engineered strains resulted to non-equimolar levels of HT, suggesting a metabolic burden although the HMS174 proved a much higher tyrosine producer than BL21-HT^{OP}.

Here by, we tried to translate the evidence to find the preferable route toward the biosynthesis of HT (monophenol or diphenol, Fig 1). When we studied each enzyme activity alone (either TYR in Fig 4 or AAS in Fig 5), there was *in-vivo* evidence that TYR was able to consume its substrates faster (tyrosine or tyrosol, within less than 2 h as compared to AAS consuming tyrosine or DOPA that lasted at least 5 h) as well as tyrosine was converted to DOPA by AAS but not at equimolar ratio (Fig 5). The abovementioned evidence suggests a delay and inefficiency in AAS activity, either alone or along TYR, that results in tyrosol and subsequently to HT accumulation (Fig 6), even at the optimal IPTG concentration. This happened most probably because the active center of TYR was either saturated with tyrosine or with DOPA and/or HT produced from the red path, causing the active TYR to exert diphenolase activity that may lead to partial inactivation due to the quinones produced [24, 44]. It should also be noted that under conditions that AAS and TYR were absent, there was no significant tyrosine consumption showing that its metabolism when provided in excess was due to the heterologous pathway. Thus, we conclude that when the system is not supplemented with any precursor molecule the metabolic flow starts from glucose and ends up to HT through the diphenol route (Fig 1B).

The intermediate tyrosine, DOPA and tyrosol build-up in HMS174 strain, in contrast to BL21 (Fig 7A and 7B), shows its potential as an HT over-producer that is kept low due to the presence of a bottleneck in the pathway. This further showed that the initial hypothesis for the advantage of the dual function of our proposed pathway was not quite exact as the native *E. coli* ALR appeared not to be as active as expected even though we attempted to optimize growth and induction conditions. Indeed, this limiting step was overcome by the overexpression of an alternative ALR gene that was cloned from *E. coli* and led to substantial increase to the production of HT. Indeed, the question as to whether the activity of a native *ALR* gene could be the limiting factor in the HT production, was answered by the over-expression of two *E. coli ALR* genes, the *ALR-D* and the *ALR-K* along with the HT biosynthetic genes (Fig 1B) and the two modules (Fig 1A, [29]) responsible for the tyrosine overproduction. These two ALR genes were selected based on earlier evaluation of their ability to convert similar aldehydes into alcohols [30, 45]. Interestingly, while the over-expression of *ALR-D* did not cause any further improvement of HT production, the over-expression of *ALR-K* increased the production of HT by 386% reaching a concentration of 1.02 mM (157.2 mg/L, Fig 8B). When the ALR-K was expressed in the respective BL21 strain, a reduction of 63% was estimated possibly because toxic intermediates were build-up. This means that the involvement of the overexpressed ALR-K resolves the biosynthetic bottleneck problem resulting in equimolar production levels of HT as compared to tyrosine initial levels (Fig 8B). Moreover, when ALR-K was functioning, the levels of DOPA and tyrosol remained minimal showing that the biosynthetic flow from tyrosine to HT was running properly; the endogenously produced tyrosine was subjected to the activity of the AAS (and the activity of the ALR) for the production of tyrosol and eventually to its transformation into HT through the hydroxylation of tyrosol by the TYR (monophenol route, Fig 1).

To slightly expand our thinking, we furthermore assumed that some intermediate products were consumed to alternative routes not for the production of HT. One of these routes is most likely to involve the function of *feaB*, producing the respective acid derivatives and thus removing precursors and substrates that would otherwise lead to HT production. The negative effect of *feaB* to remove intermediate metabolites away of the pathway of interest has been shown before [27, 28]. Such acid production may affect the activity of AAS and TYR, as showed by the decrease and/or accumulation of their respective products DOPA and/or tyrosol. Suggested deletion of *feaB* gene previously resulted in further increase of HT production [27].

The expression of the *ALR-K* is relieving the pathway from the effect of a possibly deleterious accumulating product. Its function is to convert at higher rates hydroxyphenyl-acetaldehyles produced to hydroxyphenyl-alcohols, such as tyrosol (monophenol route, Fig 1A) or hydroxytyrosol (diphenol route, Fig 1B). Given the fact that both parental strains (BL21 and HMS174) possessed the phenylacetaldehyde dehydrogenase gene (*feaB*) thus acting as hydroxyphenylacetic acid producers (Fig 1), led us to hypothesize that *feaB* must have a faster turn over than the natively existing ALR, which favored the conversion of HPAA and DHPAA to the respective acetic acid derivatives [30].

Further LC-HRMS analysis on scaled-up culture volume (1 L) of HMS174-TYR^{OP}-HT^{OP}-ALR-K strain detected higher HT production reaching 1.76 mM (270.8 mg/L, Fig 9). This increase in HT production may also be due to the stationary phase protein overproduction that is a fundamental capability of *E. coli* [46], which has been shown to lead to protein expression with higher protein solubility and thus higher enzyme activities [47].

In contrast to previous attempts to produce HT directly from glucose [27, 28], we utilized different gene sources encompassing different activities and different host strains to show the effect of the coexistence of di- and mono-phenol route. The final titer of this metabolic

Table 3. Attempts to metabolically engineer various types of microorganisms, documenting their heterologous production of hydroxytyrosol.

Precursor	Precursor concentration (mM)	Number of genes	Gene abbreviation: genetic source (*)	Hydroxytyrosol yield mg/L (mM)	Reference
2-Phenyl ethanol	2	1	*T4MO*: *Pseudomonas mendocina*	133 (0.863)	[48]
3,4-Dihydroxyphenyl acetic acid	10	1	*CAR*: *Nocardia iowensis*	616.6 (4)	[49]
Tyrosol	5	1	*PHEA*: *Geobacillus thermoglucosidasius*	770.8 (5)	[25]
Glucose	Added as carbon source	5	*TH*: *Mus musculus*<br>*PCD*: *Homo sapiens*<br>*DHPR*: *H. sapiens*<br>*DDC*: *Sus scrofa*<br>*TYO*: *Micrococcus luteus*<br>*ΔfeaB*	12.3 (0.09)	[27]
Glucose	Added as carbon source	2	*HpaBC*: *E. coli*<br>*AAS*: *Petroselinum crispum*<br>*ΔfeaB ΔtyrR ΔpheA*	208 (1.5)	[28]
Glucose	Added as carbon source	5	*HpaBC*: *E. coli*<br>*AAS*: *Papaver somniferum*<br>*TYO*: *Micrococcus luteus*<br>*aroG^{fbr}*: *2-dehydro-3-deoxyphosphoheptonate aldolase*<br>*tyrA^{fbr}*: *chorismate mutase/prephenate dehydrogenase*<br>*ΔfeaB ΔtyrR ΔpheA*	268.3 (1.74)	[50]
Glucose	Added as carbon source	14	*AroE*, *AroD*, *AroB^{OPT}*, *AroG**, *PpsA*, *TktA*, *TyrB*, *TyrA**, *AroC*, *AroA*, *AroL*: *E. coli*<br>*TYR*: *Rasltonia solanacearum*<br>*AAS*: *Petroselinum crispum*<br>*Yahk*: *E. coli*	270.8 (1.76)	This study
Glucose	Added as carbon source	7	*tyrA*: *E. coli*<br>*ppsA*: *E. coli*<br>*tktA*: *E. coli*<br>*aroG*: *E. coli*<br>*Aro10*: *S. cerevisiae*<br>*ADH6*: *S. cerevisiae*<br>*HpaBC*: *E. coli*<br>*ΔfeaB*	647 (4.2)	[51]

*(OPT, codon optimized, fbr, feedback resistant)

https://doi.org/10.1371/journal.pone.0212243.t003

construction was about 22 times higher than that of Satoh et al. [27] and 1.3 times higher than that of Chung et al. [28] (Table 3). The molarity of produced HT was 3.5 times less than the herein reported tyrosine production (6.23 mM) or 6.8 times less than the tyrosine reported to be produced by the tyrosine overproducer strain [29], a fact that dictates space for further optimization.

At the time of submission, two new articles drawn our attention for the biosynthesis of hydroxytyrosol in *E. coli*, authored by Li et al. [51] and Choo et al. [50]. The performance of their system is of interest due to the efficacy they achieved, especially for the case of Li et al. [51] who achieved a titer of 647 mg/L (4.2 mM, Table 3). They ended up in such a high concentration following a different approach, directing the metabolic flow from 4-hydroxyphenylpyruvate to 4-hydroxyphenylacetaldehyde, tyrosol and finally to HT through the action of a ketoacid decarboxylase, an alcohol dehydrogenase and a 4-hydroxyphenylacetic acid 3-hydroxylase, respectively. The pathway was overloaded with 4-hydroxyphenylpyruvate by the action of an aromatic-amino-acid aminotransferase. Their pathway proved more efficient in terms of HT production, utilizing four enzymes in contrast to our strategy that utilized fourteen; this may be an explanation of why they succeeded higher efficacy.

Concluding, the genetically tractable microbe *E. coli* provides a supreme platform for the combinatorial biosynthesis of plant natural products utilizing heterologous genes from various sources. Microbial production of plant natural products is a promising alternative to traditional methods. Here, we presented the numerous efforts made for the optimization of HT production directly from glucose utilizing a dual-pathway approach that drove the system to a HT concentration of 270.8 mg/L within 48 h. Though, further optimization efforts are ongoing to increase the final titers particularly, important for the entry of the system to the industrial production, such as the use of an AAS variant that present monospecific substrate activity that will be coupled with a TYR variant with less [44] or absent diphenolase activity or the chromosomal integration of the implicated genes. The latter may lead to the removal of antibiotics from the production plan that will further help to increase the HT purification efficiency.

Supporting information

S1 Table. List of primers used for gene clonings included in this study.
(DOCX)

S1 Fig. Prediction of a translocation signal peptide on the *Ralstonia solanacearum* tyrosinase (TYR) N-terminal. The PRED-TAT online software was utilized. The cleavage site was predicted between the two alanines (in bold) of AVAAD.
(TIF)

S2 Fig. The two plasmid modules named pS4 and pY3 for the reconstruction of L-tyrosine overproducing route from glucose. *AroA*, 5-enolpyruvoylshikimate 3-phosphate synthase; *AroB*, Dehydroquinate synthase; *AroC*, chorismate synthase; *AroD*, Dehydroquinate dehydratase; *AroE*, shikimate dehydrogenase; *AroG*, 3-deoxy-D-arabino-heptulosonate synthase; *AroL*, shikimate kinase; *PpsA*, phosphoenolpyruvate synthase; *TktA*, transketolase A; *TyrA*, chorismate mutase/prephenate dehydrogenase; *TyrB*, tyrosine aminotransferase; P, Promoter; O, Operator. T, Termination sequence [29].
(TIF)

S3 Fig. Tyrosinase and aromatic acetaldehyde synthase overexpression in *Escherichia coli* to certify their expression. (A) Upper part, protein expression in *Escherichia coli* to certify the tyrosinase protein expression. In the first lane total proteins from *E. coli* BL21-pRSF in non-induced conditions were loaded. In the second, total proteins from BL21-pRSF-RsTYR in non-induced conditions were loaded while in the third lane total proteins from BL21-pRSF-RsTYR in induced conditions were loaded. In the last lane the PiNK prestained protein ladder was loaded. The arrow in the protein marker helps to estimate the size of the protein band. Lower part, colorimetric assay with the protein crude extracts to assess the activity of tyrosinase as described in Material and Methods. (B) Protein expression in *E. coli* to certify the AAS expression. In the first lane the PiNK prestained protein ladder was loaded. In the second and the third lanes total proteins from BL21-pRSF-PcAAS in induced and non-induced conditions were loaded respectively. The arrows in the protein marker help to estimate the size of the expressed protein band.
(TIF)

Acknowledgments

The authors (ET, FV) are grateful to a) Prof. Jay D. Keasling (University of California, USA) for kindly providing pS4 and pY3 plasmids, and b) Prof. Nickolas Panopoulos (University of Crete) for providing DNA from the strain GMI1000 of *Ralstonia solanacearum*.

Author Contributions

Conceptualization: Emmanouil Trantas, Filippos Ververidis.

Funding acquisition: Filippos Ververidis.

Investigation: Emmanouil Trantas, Filippos Ververidis.

Methodology: Emmanouil Trantas, Eleni Navakoudis, Theofilos Pavlidis, Theodora Nikou, Maria Halabalaki, Leandros Skaltsounis, Filippos Ververidis.

Project administration: Filippos Ververidis.

Resources: Filippos Ververidis.

Supervision: Emmanouil Trantas, Filippos Ververidis.

Validation: Emmanouil Trantas, Filippos Ververidis.

Writing – original draft: Emmanouil Trantas, Filippos Ververidis.

Writing – review & editing: Emmanouil Trantas, Eleni Navakoudis, Theofilos Pavlidis, Theodora Nikou, Maria Halabalaki, Leandros Skaltsounis, Filippos Ververidis.

References

1. Zoidou E, Melliou E, Gikas E, Tsarbopoulos A, Magiatis P, Skaltsounis A-L. Identification of Throuba Thassos, a traditional Greek table olive variety, as a nutritional rich source of oleuropein. J Agric Food Chem. 2009; 58(1):46–50.
2. Bianco A, Mazzei RA, Melchioni C, Romeo G, Scarpati ML, Soriero A, et al. Microcomponents of olive oil. Part III. Glucosides of 2(3,4-dihydroxy-phenyl)ethanol. Food Chem. 1998; 63:461–4.
3. Bendini A, Cerretani L, Carrasco-Pancorbo A, Gomez-Caravaca AM, Segura-Carretero A, Fernandez-Gutierrez A, et al. Phenolic molecules in virgin olive oils: a survey of their sensory properties, health effects, antioxidant activity and analytical methods. An overview of the last decade. Molecules. 2007; 12 (8):1679–719. https://doi.org/10.3390/12081679 PMID: 17960082.
4. Agalias A, Magiatis P, Skaltsounis AL, Mikros E, Tsarbopoulos A, Gikas E, et al. A new process for the management of olive oil mill waste water and recovery of natural antioxidants. J Agric Food Chem. 2007; 55(7):2671–6. Epub 2007/03/14. https://doi.org/10.1021/jf063091d PMID: 17348673.
5. Fernández-Bolaños JG, López Ó, López-García MÁ, Marset A. Biological properties of hydroxytyrosol and its derivatives. In: Boskou D, editor. Olive oil—Constituents, quality, health properties and bioconversions: InTech; 2012. p. 375–96.
6. Mastralexi A, Nenadis N, Tsimidou MZ. Addressing analytical requirements to support health claims on "olive oil polyphenols"(EC Regulation 432/2012). J Agric Food Chem. 2014; 62(12):2459–61. https://doi.org/10.1021/jf5005918 PMID: 24576103
7. Visioli F, Bellomo G, Galli C. Free radical-scavenging properties of olive oil polyphenols. Biochem Biophys Res Commun. 1998; 247(1):60–4. Epub 1998/06/24. https://doi.org/10.1006/bbrc.1998.8735 PMID: 9636654.
8. Carluccio MA, Siculella L, Ancora MA, Massaro M, Scoditti E, Storelli C, et al. Olive oil and red wine antioxidant polyphenols inhibit endothelial activation—Antiatherogenic properties of Mediterranean diet phytochemicals. Arterioscl Throm Vas. 2003; 23(4):622–9. https://doi.org/10.1161/01.Atv.0000062884.69432.A0 ISI:000182165100015. PMID: 12615669
9. Visioli F, Galli C, Plasmati E, Viappiani S, Hernandez A, Colombo C, et al. Olive phenol hydroxytyrosol prevents passive smoking-induced oxidative stress. Circulation. 2000; 102(18):2169–71. Epub 2000/11/01. https://doi.org/10.1161/01.cir.102.18.2169 PMID: 11056087.
10. D'Angelo S, Manna C, Migliardi V, Mazzoni O, Morrica P, Capasso G, et al. Pharmacokinetics and metabolism of hydroxytyrosol, a natural antioxidant from olive oil. Drug Metab Disposition. 2001; 29 (11):1492–8.
11. Visioli F, Poli A, Gall C. Antioxidant and other biological activities of phenols from olives and olive oil. Medicinal research reviews. 2002; 22(1):65–75. PMID: 11746176.
12. Bisignano G, Tomaino A, Lo Cascio R, Crisafi G, Uccella N, Saija A. On the *in-vitro* antimicrobial activity of oleuropein and hydroxytyrosol. J Pharm Pharmacol. 1999; 51(8):971–4. https://doi.org/10.1211/0022357991773258 PMID: 10504039.

13. Mavrakis T, Trantas E, Agalias A, Skaltsounis L, Ververidis F, editors. Isolation of natural plant antioxidant substances from olive and katsigaros and their exploitation in plant protection. Phytopathol Mediterr; 2006.

14. Tuck KL, Hayball PJ. Major phenolic compounds in olive oil: metabolism and health effects. The Journal of Nutritional Biochemistry. 2002; 13(11):636–44. PMID: 12550060

15. Zoric N, Horvat I, Kopjar N, Vucemilovic A, Kremer D, Tomic S, et al. Hydroxytyrosol expresses antifungal activity *in vitro*. Curr Drug Targets. 2013; 14(9):992–8. Epub 2013/06/01. https://doi.org/10.2174/13894501113149990167 PMID: 23721186.

16. Mougiou N, Trikka F, Trantas E, Ververidis F, Makris A, Argiriou A, et al. Expression of hydroxytyrosol and oleuropein biosynthetic genes are correlated with metabolite accumulation during fruit development in olive, *Olea europaea*, cv. Koroneiki. Plant Physiol Biochem. 2018; 128:41–9. https://doi.org/10.1016/j.plaphy.2018.05.004 PMID: 29753981

17. Alagna F, Mariotti R, Panara F, Caporali S, Urbani S, Veneziani G, et al. Olive phenolic compounds: metabolic and transcriptional profiling during fruit development. BMC Plant Biol. 2012; 12:162. https://doi.org/10.1186/1471-2229-12-162 PMID: 22963618; PubMed Central PMCID: PMC3480905.

18. Owen RW, Giacosa A, Hull WE, Haubner R, Spiegelhalder B, Bartsch H. The antioxidant/anticancer potential of phenolic compounds isolated from olive oil. Eur J Cancer. 2000; 36(10):1235–47. Epub 2000/07/07. https://doi.org/10.1016/s0959-8049(00)00103-9 PMID: 10882862.

19. Owen RW, Mier W, Giacosa A, Hull WE, Spiegelhalder B, Bartsch H. Phenolic compounds and squalene in olive oils: the concentration and antioxidant potential of total phenols, simple phenols, secoiridoids, lignansand squalene. Food Chem Toxicol. 2000; 38(8):647–59. Epub 2000/07/26. https://doi.org/10.1016/s0278-6915(00)00061-2 PMID: 10908812.

20. Owen RW, Haubner R, Mier W, Giacosa A, Hull WE, Spiegelhalder B, et al. Isolation, structure elucidation and antioxidant potential of the major phenolic and flavonoid compounds in brined olive drupes. Food Chem Toxicol. 2003; 41(5):703–17. Epub 2003/03/28. https://doi.org/10.1016/s0278-6915(03)00011-5 PMID: 12659724.

21. Servili M, Selvaggini R, Esposto S, Taticchi A, Montedoro G, Morozzi G. Health and sensory properties of virgin olive oil hydrophilic phenols: agronomic and technological aspects of production that affect their occurrence in the oil. J Chromatogr. 2004; 1054(1–2):113–27.

22. Capasso R, Evidente A, Avolio S, Solla F. A highly convenient synthesis of hydroxytyrosol and its recovery from agricultural waste waters. J Agric Food Chem. 1999; 47(4):1745–8. https://doi.org/10.1021/jf9809030 PMID: 10564048.

23. Zhang Z-L, Chen J, Xu Q, Rao C, Qiao C. Efficient synthesis of hydroxytyrosol from 3,4-dihydroxybenzaldehyde. Synthetic Communications. 2012; 42(6):794–8. https://doi.org/10.1080/00397911.2010.531369

24. Espin JC, Soler-Rivas C, Cantos E, Tomas-Barberan FA, Wichers HJ. Synthesis of the antioxidant hydroxytyrosol using tyrosinase as biocatalyst. J Agric Food Chem. 2001; 49(3):1187–93. https://doi.org/10.1021/jf001258b PMID: 11312833

25. Orenes-Piñero E, García-Carmona F, Sánchez-Ferrer Á. A new process for obtaining hydroxytyrosol using transformed *Escherichia coli* whole cells with phenol hydroxylase gene from *Geobacillus thermoglucosidasius*. Food Chem. 2013; 139(1–4):377–83. https://doi.org/10.1016/j.foodchem.2012.12.063 PMID: 23561120

26. Allouche N, Damak M, Ellouz R, Sayadi S. Use of whole cells of *Pseudomonas aeruginosa* for synthesis of the antioxidant hydroxytyrosol via conversion of tyrosol. Appl Environ Microbiol. 2004; 70(4):2105–9. https://doi.org/10.1128/AEM.70.4.2105-2109.2004 PMID: 15066802.

27. Satoh Y, Tajima K, Munekata M, Keasling JD, Lee TS. Engineering of L-tyrosine oxidation in *Escherichia coli* and microbial production of hydroxytyrosol. Metab Eng. 2012; 14(6):603–10. Epub 2012/09/06. https://doi.org/10.1016/j.ymben.2012.08.002 PMID: 22948011.

28. Chung D, Kim SY, Ahn J-H. Production of three phenylethanoids, tyrosol, hydroxytyrosol, and salidroside, using plant genes expressing in *Escherichia coli*. Scientific reports. 2017;7. https://doi.org/10.1038/s41598-017-00035-9

29. Juminaga D, Baidoo EE, Redding-Johanson AM, Batth TS, Burd H, Mukhopadhyay A, et al. Modular engineering of L-tyrosine production in Escherichia coli. Appl Environ Microbiol. 2012; 78(1):89–98. https://doi.org/10.1128/AEM.06017-11 PMID: 22020510; PubMed Central PMCID: PMC3255607.

30. Koma D, Yamanaka H, Moriyoshi K, Ohmoto T, Sakai K. Production of aromatic compounds by metabolically engineered *Escherichia coli* with an expanded shikimate pathway. Appl Environ Microbiol. 2012; 78(17):6203–16. https://doi.org/10.1128/AEM.01148-12 PMID: 22752168; PubMed Central PMCID: PMC3416637.

31. Torrens-Spence MP, Gillaspy G, Zhao B, Harich K, White RH, Li J. Biochemical evaluation of a parsley tyrosine decarboxylase results in a novel 4-hydroxyphenylacetaldehyde synthase enzyme. Biochem

Biophys Res Commun. 2012; 418(2):211–6. Epub 2012/01/24. https://doi.org/10.1016/j.bbrc.2011.12.124 PMID: 22266321.

32. Hernandez-Romero D, Sanchez-Amat A, Solano F. A tyrosinase with an abnormally high tyrosine hydroxylase/dopa oxidase ratio. FEBS J. 2006; 273(2):257–70. Epub 2006/01/13. https://doi.org/10.1111/j.1742-4658.2005.05038.x PMID: 16403014.
33. Schomburg I, Chang A, Ebeling C, Gremse M, Heldt C, Huhn G, et al. BRENDA, the enzyme database: updates and major new developments. Nucleic Acids Res. 2004; 32(suppl 1):D431–D3.
34. Marisch K, Bayer K, Cserjan-Puschmann M, Luchner M, Striedner G. Evaluation of three industrial Escherichia coli strains in fed-batch cultivations during high-level SOD protein production. Microb Cell Fact. 2013; 12(1):58. https://doi.org/10.1186/1475-2859-12-58 PMID: 23758670
35. Bagos PG, Nikolaou EP, Liakopoulos TD, Tsirigos KD. Combined prediction of Tat and Sec signal peptides with hidden Markov models. Bioinformatics. 2010; 26(22):2811–7. https://doi.org/10.1093/bioinformatics/btq530 PMID: 20847219
36. Kawalleck P, Keller H, Hahlbrock K, Scheel D, Somssich IE. A pathogen-responsive gene of parsley encodes tyrosine decarboxylase. J Biol Chem. 1993; 268(3):2189–94. Epub 1993/01/25. PMID: 8420986.
37. Facchini PJ, Penzes-Yost C, Samanani N, Kowalchuk B. Expression patterns conferred by tyrosine/dihydroxyphenylalanine decarboxylase promoters from opium poppy are conserved in transgenic tobacco. Plant Physiol. 1998; 118(1):69–81. Epub 1998/09/11. https://doi.org/10.1104/pp.118.1.69 PMID: 9733527; PubMed Central PMCID: PMC34875.
38. Hernandez-Romero D, Solano F, Sanchez-Amat A. Polyphenol oxidase activity expression in *Ralstonia solanacearum*. Appl Environ Microbiol. 2005; 71(11):6808–15. Epub 2005/11/05. https://doi.org/10.1128/AEM.71.11.6808-6815.2005 PMID: 16269713; PubMed Central PMCID: PMC1287666.
39. Bradford MM. A rapid and sensitive method for the quantitation of microgram quantities of protein utilizing the principle of protein-dye binding. Anal Biochem. 1976; 72(1–2):248–54.
40. Schaerlaekens K, Schierova M, Lammertyn E, Geukens N, Anne J, Van Mellaert L. Twin-arginine translocation pathway in *Streptomyces lividans*. J Bacteriol. 2001; 183(23):6727–32. https://doi.org/10.1128/JB.183.23.6727-6732.2001 PMID: 11698358; PubMed Central PMCID: PMC95510.
41. Kouloura E, Skaltsounis AL, Michel S, Halabalaki M. Ion tree-based structure elucidation of acetophenone dimers (AtA) from Acronychia pedunculata and their identification in extracts by liquid chromatography electrospray ionization LTQ-Orbitrap mass spectrometry. J Mass Spectrom. 2015; 50(3):495–512. https://doi.org/10.1002/jms.3556 PMID: 25800186.
42. Ververidis F, Trantas E, Douglas C, Vollmer G, Kretzschmar G, Panopoulos N. Biotechnology of flavonoids and other phenylpropanoid-derived natural products. Part I: Chemical diversity, impacts on plant biology and human health. Biotechnol J. 2007; 2(10):1214–34. https://doi.org/10.1002/biot.200700084 PMID: 17935117.
43. Trantas E, Panopoulos N, Ververidis F. Metabolic engineering of the complete pathway leading to heterologous biosynthesis of various flavonoids and stilbenoids in *Saccharomyces cerevisiae*. Metab Eng. 2009; 11(6):355–66. https://doi.org/10.1016/j.ymben.2009.07.004 PMID: 19631278
44. Molloy S, Nikodinovic-Runic J, Martin LB, Hartmann H, Solano F, Decker H, et al. Engineering of a bacterial tyrosinase for improved catalytic efficiency towards D-tyrosine using random and site directed mutagenesis approaches. Biotechnol Bioeng. 2013; 110(7):1849–57. https://doi.org/10.1002/bit.24859 PMID: 23381872.
45. Rodriguez GM, Atsumi S. Toward aldehyde and alkane production by removing aldehyde reductase activity in *Escherichia coli*. Metab Eng. 2014; 25:227–37. https://doi.org/10.1016/j.ymben.2014.07.012 PMID: 25108218; PubMed Central PMCID: PMC4411948.
46. Ou J, Wang L, Ding X, Du J, Zhang Y, Chen H, et al. Stationary phase protein overproduction is a fundamental capability of *Escherichia coli*. Biochem Biophys Res Commun. 2004; 314(1):174–80. https://doi.org/10.1016/j.bbrc.2003.12.077 PMID: 14715262
47. Galloway CA, Sowden MP, Smith HC. Increasing the yield of soluble recombinant protein expressed in *E. coli* by induction during late log phase. BioTechniques. 2003; 34(3):524–6, 8, 30. https://doi.org/10.2144/03343st04 PMID: 12661158
48. Brouk M, Fishman A. Improving process conditions of hydroxytyrosol synthesis by toluene-4-monooxygenase. Journal of Molecular Catalysis B: Enzymatic. 2012; 84:121–7.
49. Napora-Wijata K, Strohmeier GA, Winkler M. Biocatalytic reduction of carboxylic acids. Biotechnology journal. 2014; 9(6):822–43. Epub 2014/04/17. https://doi.org/10.1002/biot.201400012 PMID: 24737783.
50. Choo HJ, Kim EJ, Kim SY, Lee Y, Kim B-G, Ahn J-H. Microbial synthesis of hydroxytyrosol and hydroxysalidroside. Appl Biol Chem. 2018; 61(3):295–301. https://doi.org/10.1007/s13765-018-0360-x
51. Li X, Chen Z, Wu Y, Yan Y, Sun X, Yuan Q. Establishing an artificial pathway for efficient biosynthesis of hydroxytyrosol. ACS Synth Biol. 2018; 7(2):647–54. https://doi.org/10.1021/acssynbio.7b00385 PMID: 29281883.
